# Drug-induced diabetes type 2: *In silico* study involving class B GPCRs

**DOI:** 10.1371/journal.pone.0208892

**Published:** 2019-01-16

**Authors:** Dorota Latek, Ewelina Rutkowska, Szymon Niewieczerzal, Judyta Cielecka-Piontek

**Affiliations:** 1 Faculty of Chemistry, University of Warsaw, Warsaw, Poland; 2 Department of Pharmacognosy, Faculty of Pharmacy, Poznan University of Medical Sciences, Poznan, Poland; Indian Institute of Technology Kanpur, INDIA

## Abstract

A disturbance of glucose homeostasis leading to type 2 diabetes mellitus (T2DM) is one of the severe side effects that may occur during a prolonged use of many drugs currently available on the market. In this manuscript we describe the most common cases of drug-induced T2DM, discuss available pharmacotherapies and propose new ones. Among various pharmacotherapies of T2DM, incretin therapies have recently focused attention due to the newly determined crystal structure of incretin hormone receptor GLP1R. Incretin hormone receptors: GLP1R and GIPR together with the glucagon receptor GCGR regulate food intake and insulin and glucose secretion. Our study showed that incretin hormone receptors, named also gut hormone receptors as they are expressed in the gastrointestinal tract, could potentially act as unintended targets (off-targets) for orally administrated drugs. Such off-target interactions, depending on their effect on the receptor (stimulation or inhibition), could be beneficial, like in the case of incretin mimetics, or unwanted if they cause, e.g., decreased insulin secretion. In this *in silico* study we examined which well-known pharmaceuticals could potentially interact with gut hormone receptors in the off-target way. We observed that drugs with the strongest binding affinity for gut hormone receptors were also reported in the medical information resources as the least disturbing the glucose homeostasis among all drugs in their class. We suggested that those strongly binding molecules could potentially stimulate GIPR and GLP1R and/or inhibit GCGR which could lead to increased insulin secretion and decreased hepatic glucose production. Such positive effect on the glucose homeostasis could compensate for other, adverse effects of pharmacotherapy which lead to drug-induced T2DM. In addition, we also described several top hits as potential substitutes of peptidic incretin mimetics which were discovered in the drug repositioning screen using gut hormone receptors structures against the ZINC15 compounds subset.

## Introduction

Since 1980 the number of people living with diabetes has nearly quadrupled according to World Health Organization [1]. The most predominant form of diabetes is type 2 diabetes mellitus (T2DM) which starts from developing insulin resistance and usually relative (rather than absolute) insulin deficiency. It occurs more frequently in women with prior gestational diabetes mellitus and in individuals with hypertension or dyslipidemia. Interestingly, its frequency and success in pharmacotherapy varies in different ethnic subgroups [2, 3]. Another, often neglected, reason is pharmacotherapy of chronic diseases [4]. The risk of developing T2DM increases with age, obesity and lack of physical activity. Its prevalence among older people is bringing interest from public health-care managements for necessity of benefit-risk judgments [5].

Homeostasis of glucose serum levels can be disturbed by pharmacotherapy in three major areas: pancreas, liver and peripheral tissues which are associated with glucose and insulin production and secretion [4]. Growing data in the genomics and metabolomics fields of research provided evidence that occurring of particular SNPs (single nucleotide polymorphisms) and particular amino acids in the baseline plasma metabolite level can be associated with the increased risk for drug-induced diabetes [6]. On the other hand, particular drug classes, e.g., glucocorticosteroids, statins, diuretics and beta-blockers [3, 4] may induce diabetes type 2 more frequently than the other drug classes due to their influence on the hepatic glucose production, pancreatic insulin secretion and peripheral tissues insulin sensitivity [7]. The complex molecular mechanism of drug-induced T2DM varies from one drug class to another (e.g. beta-blockers [3] vs. steroids [8]) and still is not fully understood because there are many pathways involved in insulin secretion which could be directly or indirectly affected by a given drug [8]. In general, one of the major causes of side effects such as T2DM is a weak selectivity of drugs resulting in occurrence of the off-target interactions [9, 10]. Such off-target interactions may not necessarily involve an original drug but, for example, its active metabolites [11, 12]. A notable example of the experimentally confirmed off-target interaction leading to hyperglycemia was detected between simvastatin, which on-target is HMG-CoA reductase, and L-type Ca2+ channels [8]. Both, on and off-targets of simvastatin are alpha-helical transmembrane proteins but they differ in localization (endoplasmic reticulum vs. cellular membrane). The location of the intended molecular target (on-target) of a given drug in particular body tissues and organs (gut, liver, pancreas, CNS, blood vessels) is an important premise to trace its unintended targets (off-targets) and prevent associated side effects [13, 14]. For instance, a change of molecular targets from salt transporters to urea transporters which are expressed specifically in kidneys [15] could be a way to minimize side effects of diuretics [15]. Another well-known examples of avoiding side effects in the field of pharmacotherapy of hypertension are: an older study on angiotensin receptor blockers [16, 17] and a more recent study on SPAK kinase inhibitors [17].

There are also other ways to deal with drug-induced diabetes and other drug side effects, for example, a polytherapy. Any polytherapy can be optimized in the direction of side effects elimination caused by selected groups of drugs, by adding another ‘protecting’ drug to the therapy, e.g., mentioned above angiotensin receptor blockers to hydrochlorohiazide [18] or a potassium supplement to chlortalidone (thiazide analog) [19]. Both mentioned examples of polytherapy protect patients from developing new-onset diabetes while treating hypertension. Also, broadly understood SAR (structure-activity relationship) studies lead to the introduction of more pharmacologically effective analogs with much milder side effects, also with regard to drug-induced T2DM, e.g., beta-1 selective blockers vs. old beta-blockers [9, 20]. Pharmacophore modeling, QSAR studies, ADME/ADME-Tox properties prediction and other in-silico methods for SAR (structure-activity relationship) determination are standard approaches to improve drug selectivity not only for the sake of its efficacy but also to reduce its off-target or toxic effects [21, 22]. For example, in a recent study [23] the molecular docking was used to discriminate compounds which caused adverse drug reactions involving HLA surface proteins.

In the current work, we proposed the *in silico* solution to drug-induced diabetes problem which, e.g., could help to select the least harmful pharmacotherapies for diabetics. Our solution was based on the positive incretin effect which could be enhanced by drugs in the off-target manner and which could compensate for their negative effect on the glucose homeostasis in other metabolic pathways. Namely, for several commonly used drug classes, we gathered medical information on drug-induced diabetes available in the literature and also deposited in SIDER (Side Effect Resource) [24] and convert it, where possible, into T2DM-related drug rankings inside each drug class. Then, we selected the compounds library which included those drug classes (ZINC15 FDA-approved drugs) and carried out the virtual screening (VS) study against that library using structures of incretin receptors. We limited all known glucose homeostasis-disruptors among drugs to such drug classes for which the relative diabetogenic effect among their members was reported in details and thus the T2DM-related ranking for those drug classes could be easily prepared. Although our results are preliminary, we observed a correlation between T2DM-related drug rankings and drug-receptor binding affinities obtained from VS. Based on that, we managed to accurately nominate the least harmful drugs for the glucose homoeostasis in each drug class from four commonly used drug classes (beta-blockers [25], statins, diuretics, steroids). The most accurate predictions were obtained for the beta-blockers drug class and were described in the separate manuscript [25] that is complementary to the current study. Briefly, in that manuscript, we hypothesized that the new generation beta-blockers, observed clinically as less T2DM-inducing than the old generation, could owe that advantage, at least partially, to the compensating incretin effect enhanced by their off-target interactions with gut hormone receptors. In the current manuscript we described similar observations made for other drug classes of known glucose homeostasis disruptors. Especially, in the case of statins, we managed to accurately select the least harmful drugs in that drug class which could owe that effect, at least partially, to potential off-target interactions with gut hormone receptors.

We selected gut hormone receptors for the purpose of the current study for two reasons. One reason is their major role in the glucose metabolism (see below). The other reason is their expression pattern. Gut hormone receptors are expressed in the gastrointestinal tract where also orally administrated drugs are absorbed to the circulatory. It was confirmed, e.g., in [13] that tissue expression and cellular localization of the potential off-target proteins play crucial role in occurrence of the associated drug side effects. That is why we believe that both, function and localization of gut hormone receptors are factors which make our computational study on the drug off-target interactions plausible.

Gut hormone receptors, also known as glucagon receptors, constitute of three G protein-coupled receptors (GPCRs) from the class B (secretin-like) receptors: glucagon receptor (GCGR), glucagon-like peptide 1 receptor (GLP1R) and gastric inhibitory polypeptide receptor (GIPR). Only two of them (GIPR and GLP1R) are named incretin hormone receptors. However, it is common in the literature to refer the incretin effect not only to the stimulation of GIPR and GLP1R but also to the inhibition of GCGR. Endogenous peptide ligands of GIPR, GLP1R and GCGR regulate glucose homeostasis and affect insulin secretion [26, 27]. GCGR, GIPR and GLP1R are expressed mainly in digestive system (pancreatic beta-cells, intestine, liver and kidney) and also in circulatory (heart, blood vessels) [28], which is the target system of, e.g., beta-blockers. Thus, the first response of the central nervous system (CNS) to glucose is observed at the gut level, prior to absorption to the circulation [29]. Moreover, GLP1R receptors which are expressed in brain activate hypothalamus providing negative feedback when sensing the oral nutrient overload and thus play a role in appetite regulation and reducing the body weight [26]. As we mentioned above, such gastrointestinal localization of incretin hormone receptors might favor off-target interactions with orally administrated drugs. For example, it was confirmed in mouse and later in human studies that orally administrated metformin can influence the gut-brain axis by stimulating off-target interactions with the incretin receptor GLP1R [30].

As for the use in pharmacotherapy, incretin hormones GIP and GLP have focused attention a long time ago during the study on variation of insulin secretion depending on the glucose administration route (food vs. injection) [28]. Nevertheless, the first peptidic GLP1R agonist to be used as a drug was approved by FDA in 2005 (exenatide—Byetta) [26] and still the number of small-molecule non-peptidic compounds targeting incretin hormone receptors to treat diabetes and obesity is extremely small [28]. Only recently, Food and Drug Administration and European Medicines Agency have issued an assessment on the incretin-based therapeutics [31]. Thus, incretin therapies became the second or third line treatment options for diabetes type 2 [32]. Unfortunately, currently available agonists are peptides (GLP or GIP analogs) of low bioavailability and half-life [33] and potentially may increase other side effects themselves [31, 34]. Therefore, discovering of small molecule non-peptidic ligands is of high interest [35]. Progress in crystallography of the class B GPCRs [36–41] will undoubtedly raise the number of drug discovery studies targeting that class of receptors for the next years [28, 33]. As for the mechanism of action which is the basis of incretin therapies, the increase in GIP and GLP concentration positively affects blood insulin levels, while, on the contrary glucagon increases hepatic production of glucose [42, 43]. Importantly, GIP stimulates also pancreatic beta-cell proliferation and prevents beta-cell apoptosis. Both effects, from incretin hormones and glucagon were used to develop anti-diabetes agents: GLP1R and GIPR agonists [44, 45] or antagonists [26] and GCGR antagonists [46, 47]. Recently, a dual agonist for GCGR and GLP1R [48] and a triple-action agonist (triagonist) for all three receptors enhancing the incretin effect and simultaneously acting as a buffer for diabetogenic effect of glucagon has been developed [49, 50]. Developing new small-molecule ligands for incretin hormone receptors can be carried out by drug discovery or drug repurposing. We decided to extend the current study on off-target interactions by the latter approach. Namely, the second part of our work was dedicated to the drug repositioning (or drug repurposing) concept. According to [51, 52] drug repositioning is simply finding new uses for existing drugs. That phenomenon is based on the observation of common molecular pathways for different active pharmaceutical ingredients (APIs). It is widely known that the occurrence of side effects gives indications for the use of a particular API in different pathological units than the one for which clinical trials were originally conducted. There are many APIs [51], for which a new functionality was defined based on the observed adverse reactions. For example, thalidomide mutagenic effect was used for treatment of cancer. In order to find new APIs to use in incretin therapies we carried out the virtual screening study using gut hormone receptors structures and the limited ZINC15 subset which included drugs already available on the market (FDA-approved drugs). As a result, several pharmaceuticals were proposed as potential substitutes of incretin peptides mimetics in the incretin therapies.

We decided to use virtual screening as a strategy for drug repositioning because of its documented usefulness in drug discovery and lead optimization studies described, e.g., in the recent manuscript of D. Rognan [53]. In general, virtual screening methods can be very effective when the experimental knowledge is combined with a proper computational approach. The critical analysis of VS results reports the average hit rates of ca. 13% and that is much higher than for typical experimental HTS hit rates (0.01%–0.14%) [54]. An example of a successful *in silico* approach was described in [55]. Docking of the lead-like ZINC subset with about 1 million compounds to a crystal structure of the β2-adrenergic receptor resulted in the final selection of 25 compounds based only on the high docking score. The following experimental analysis of those compounds showed that six of them were indeed bound to the receptor and one compound exhibited an excellent potency with Ki equal to 9 nM. That provided an excellent hit rate of 24%. In another study [56], authors docked a library of over 3 million compounds against the μ-opioid receptor structure and identified new scaffolds unrelated to known opioids. Less than 50 tested compounds were selected for further analysis and a final lead molecule—PZM21, an agonist with potency and efficacy similar to morphine, was discovered. Tested in mouse hotplate assays, PZM21 showed reduced adverse effects and, however still in the basic research, is considered as a possible first effective and safe opioid pain-killer. Above examples reflect that VS methods can be a powerful tool in computer-aided drug discovery.

## Results and discussion

### Structural features of incretin hormone GPCRs

**Fig 1:**
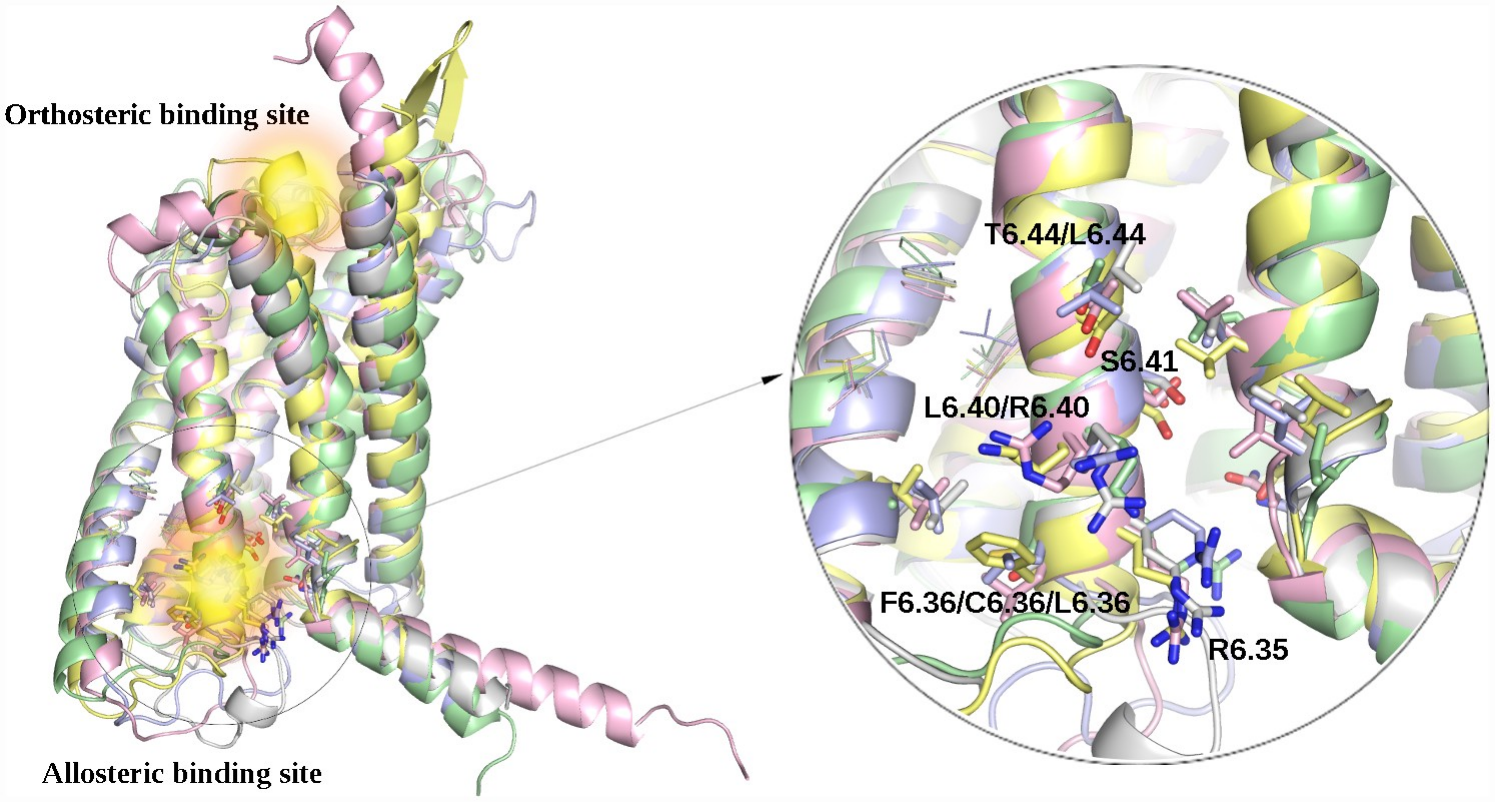
Structural comparison of the selected class B GPCRs. Yellow—GCGR (5XEZ), pink—GIPR (4L6R), green—GLP1R (5VEW). Two binding sites (orthosteric and allosteric) were depicted as yellow spheres. The binding site for allosteric modulators of the selected class B GPCRs was shown in details on the right. The figure was prepared with Pymol [131].

Available to date crystal structures of gut hormone GPCRs from class B (see Table 1 and Fig 1) showed a few distinct features of those receptors compared to class A GPCRs. Namely, N-terminal helix is longer than in the case of the class A GPCRs and with a linker, called a stalk, is joined with extracellular domain (ECD) binding peptide hormones. A long extracellular loop 1 (EC1) is involved in the peptide binding. The orthosteric peptide binding site surrounded by EC loops is much more spacious than that in the class A while containing the same disulphide bond connecting EC2 with transmembrane helix 3 (TMH3). The most distinct feature of class B GPCRs is the localization of the additional allosteric binding site between TMH6 and TMH7 that faces the lipid bilayer [37]. The major determinant of the negative allosteric modulation (NAM) selectivity between GLP1R and GCGR is C/F6.36 [40] (see Fig 1). C6.36 in GLP1R has been identified to be crucial for covalent interactions with positive allosteric modulators (PAMs) [40]. In the current study, we performed docking to both, orthosteric and allosteric binding site of the selected class B GPCRs. Mechanism of the class B activation involves moving away the intracellular part of TMH6 from the receptor center and the clockwise rotation of the extracellular ends of TMH1, TMH6 and TMH7 as showed in the partly activated 5NX2 structure of GLP1R [39]. The cryo-EM active structure of GLP1R (PDB id: 5VAI) [57] revealed also the extended extracellular end of TMH2 (with shorter ECL1) to form interactions with a peptide.

**Table 1:** Template structures used for the receptor model building and model quality assessment (partly adapted from [25]).

Receptor	Domain, ligand	Conformational state	PDB id	PDB structure modifications	GPCR models build on this template
GCGR	TMD	inactive	4L6R	MD (only for GCGR and GIPR)	GCGR, GIPR, GLP1R
GCGR	TMD	inactive	5EE7	MD (only for GCGR and GIPR)	GCGR, GIPR, GLP1R
GCGR	TMD	inactive	5XEZ	MD (only for GCGR)	GCGR
GCGR	TMD, ECD, Glucagon	active	MD simulation based on 4L6R and 4ERS	Seven N-terminal residues of glucagon truncated	Filtering based on steric hindrances—GCGR, GIPR, GLP1R
GLP1R	TMD, ECD	active	5NX2	MD	GLP1R
GLP1R	TMD	active	5VAI	MD	GLP1R
GLP1R	TMD	inactive	5VEW	MD	GLP1R
GLP1R	TMD	inactive	5VEX	MD	GLP1R
GLP1R	ECD, GLP	active	3IOL	Three N-terminal residues of GLP were truncated	Filtering based on steric hindrances—GLP1R
GIPR	ECD, GIP	active	2QKH	Last six residues of GIP were truncated	Filtering based on steric hindrances—GIPR

In Fig 1, we showed representative examples of GPCR models used in the current study. Two possible binding sites were depicted. The first one was the orthosteric binding site located inside the transmembrane domain but facing the extracellular space. The second one is the allosteric binding site between helices TM5-7 that faces the interior of the lipid bilayer. On the right panel we presented in details residues in the area of the allosteric binding site. Arg6.35 (residue numbering fit Hollenstein et al. [58]) and Asn7.61 are conserved among all studied class B GPCRs. In the GLP1R structure Arg6.35 forms a hydrogen bond with a side chain of Asn8.47 located at the beginning of the helix H8. Residues important for the ligand binding (see 5XEZ and 5XF1): Leu7.56, Lys6.40, Ser6.40 and Thr6.44 are also conserved in the glucagon GPCRs. The mentioned above residue Phe6.36, associated with selectivity of allosteric modulation, which is located at the N-terminal end of TMH6, is substituted with Leu (GIPR) and Cys (GLP1R). Leu5.65 at the C-terminal end of TMH5 is conserved among the selected class B GPCRs (the LxxL motif [59]).

### Diabetogenic and potential incretin effects demonstrated by selected drug classes

Although relative diabetogenic effects of drugs inside the commonly used drug classes are studied at least to the minor extent in several cases (see Tables B and C in S1 File), there is only few studies comparing drug classes to each other such as, e.g., Elliot et al. [60] or [61], where beta-blockers were compared to diuretics. In those studies, it was emphasized that such information is crucial for selection the least harmful pharmacotherapy of hypertension in diabetic patients. In our study we proposed the *in silico* approach for performing such selection.

**Fig 2:**
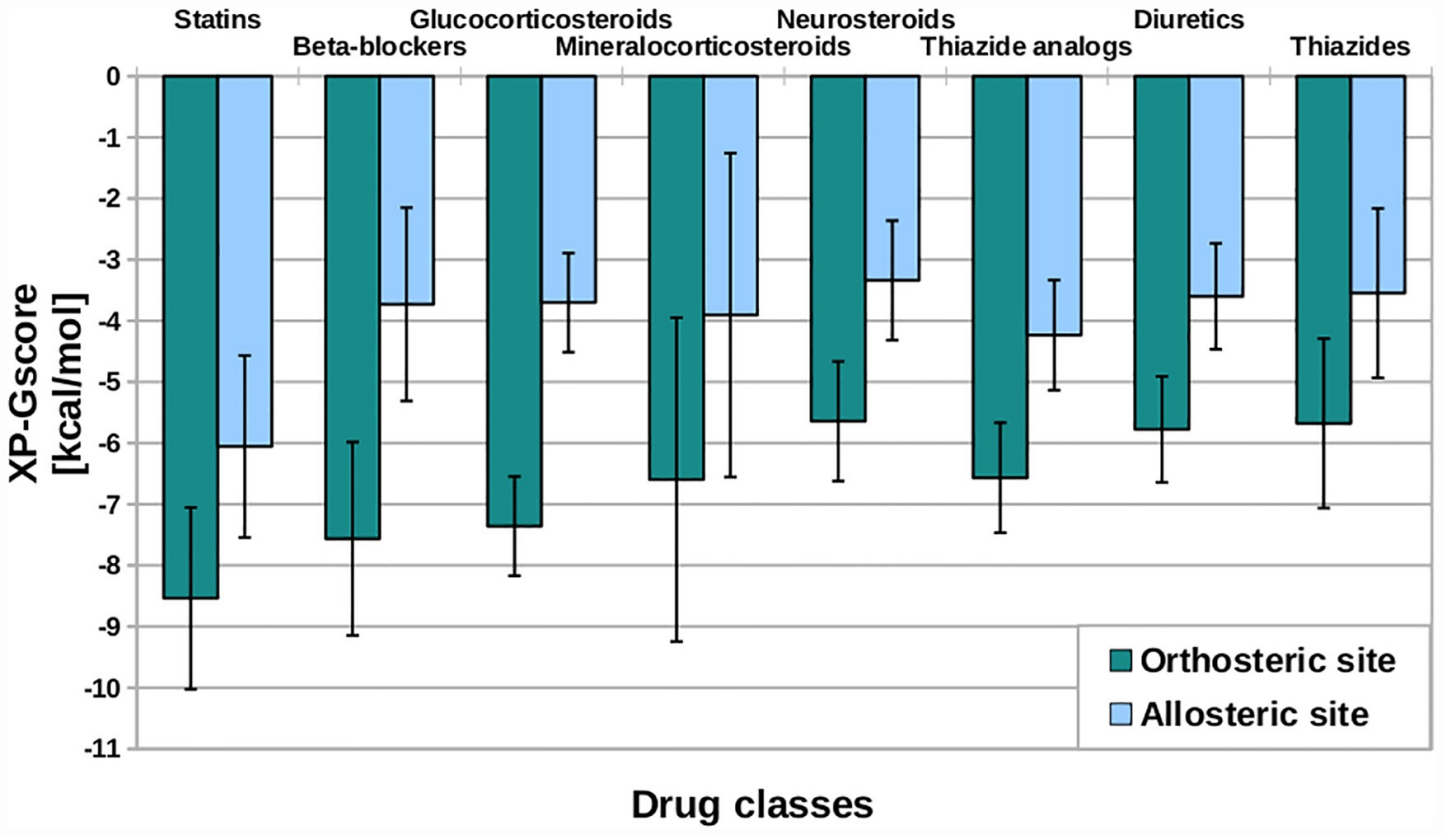
VS results for the selected drug classes. Here, we presented average values of XP-GScores obtained from virtual screening against all gut hormone receptors structures. We indicated error bars equal to standard deviations.

Namely, based on [62–64] we gathered various drug classes which are known to disrupt glucose metabolism (see Fig 2). Then, we performed the VS study using structures of gut hormone receptors against the compounds library containing those drug classes and computed the average XP-GScore for all drugs from each class (see Fig 2). Those average XP-GScores reflected the average strength of the binding affinity of the particular drug class for gut hormone receptors. We suspected that, drug classes (neurosteroids and thiazides) with the weakest theoretical binding affinity for the selected GPCRs most probably do not exhibit any off-target interactions with them. On the other hand, drug classes (beta-blockers and statins) which were of the strongest theoretical binding affinity for the gut hormone GPCRs may indeed bind to them in the off-target manner and thus may enhance the positive incretin effect on the glucose homeostasis which could compensate for their negative influence in other metabolic pathways. Nevertheless, experimental studies are certainly needed to confirm that.

Among selected drug classes, thiazides [65] and glucocorticosteroids are the most frequently mentioned in the literature as associated with drug-induced T2DM [66]. In our study, among all included steroid-like drugs, glucocorticosteroids were bound with quite strong affinity (low absolute values of XP-GScore) to gut hormone GPCRs (see Fig 2). In contrast, the weakest binding affinity for gut hormone GPCRs, not only among steroids but also among all included drug classes, showed neurosteroids. Mineralocorticosteroids showed the average binding affinity for gut hormone GPCRs. We did not include detailed results for every steroid drug like in the case of statins (see **Diabetogenic and potential incretin effects demonstrated by statins**). The reason for this is that for steroids we could not find sufficient medical information to compose the T2DM-related ranking describing their relative influence on the glucose serum levels. Nevertheless, among glucocorticosteroids, deflazacort (see Table D in S1 File) is frequently mentioned to be the low-risk drug for diabetics [67] and indeed exhibited quite strong binding affinity for gut hormone GPCRs in our study (data not shown). The exact mechanism of steroid-induced diabetes or hyperglycemia is not fully understood but it is highly correlated with therapeutic doses [68–70]. In general, glucocorticosteroids increase insulin resistance and glucose intolerance and simultaneously inhibit pancreatic insulin production by inducing β-cell dysfunction [66]. Interestingly, there are studies on treatment of steroid-induced diabetes with incretins [71]. Depending on the type, steroids bind to different molecular targets: nuclear transcription factors such as glucocorticoid receptor (GR) and mineralocorticoid receptor (MR) and cell surface receptors such as ion channels and GPCRs (neurosteroids). Recently, a novel interaction between beta-arrestin signaling and glucocorticosteroid receptor (GR) has been reported [72]. Nevertheless, due to a difference in the cell localization of GPCRs and GR, we have not found any literature data confirming direct interactions of gluco- and mineralocorticosteroids with TMD or ECD domains of GPCRs, though it was reported that they can suppress GLP-1 secretion [73]. It is possible, that strong binding affinities observed in our VS study were only a result of structural similarities between binding sites of GR or MR and GPCRs. The case of curcumin [74], a false positive in many virtual screenings for anti-diabetes drugs (a so-called pan-assay interference compound—PAIN), shows the need for a critical approach to VS results. Nevertheless, there is a case of endogenous hormone estrogen (also classified as a steroid), which binds to both, nuclear estrogen receptor (ER) and the membrane GPCR named GPER1 [75]. Certainly, experimental studies are needed to confirm if any off-target interaction between glucocorticosteroids and incretin hormone receptors takes place.

Thiazides, weakly bound to the gut hormone receptors in our study (see Fig 2) are known to induce diabetes the most among diuretics drug class [76] (see Table C in S1 File based on the currently available medical information [77–80]). The main drug targets for thiazides are cell membrane proteins: Na^+^/Cl^-^ symporter and Na^+^/Ca^2+^ antiporter. Other diuretics mentioned in the current study target Na^+^-K^+^-2Cl^-^ symporter (loop diuretics), metalloenzyme carbonic anhydrase (carbonic anhydrase inhibitors), sodium channel (sodium channel blockers) or compete with aldosterone (e.g., spironolactone). The mechanism of diuretic-induced diabetes is complex and not fully understood at the molecular level but certainly involves many cell signaling pathways [81, 82]. Among all known diuretics subclasses, except thiazides, also loop diuretics were associated with the increased risk of drug-induced T2DM [79]. On the other hand, the smallest diabetogenic effect was reported for carbonic anhydrase inhibitors and potassium-sparing diuretics (see Table C in S1 File) [83, 84]. Also, the least influence on the glucose serum level was observed for analogs of thiazides recently introduced to the market [80, 85–87]. In our study, analogs of thiazides indeed exhibited relatively strong binding affinity for all gut hormone GPCRs on average (see Fig 2) comparing the basic drug class of thiazides. We believe that chemical modifications introduced to analogs of thiazides comparing thiazides in order to minimize their side effects could also result in their increased binding towards incretin hormone receptors leading to indeed minimized risk of T2DM observed in clinical trials.

As we mentioned above, statins and beta-blockers showed the highest binding affinity for all gut hormone GPCRs, among all tested drug classes. Interestingly, the recently discovered beta-blocker—Compound no. 15 [88] was ranked relatively high in all carried out virtual screenings which was described in our second manuscript [25] that is complementary to the current one. We believe that such off-target interactions are possible due to the fact, that beta-blockers’ on-targets belong to the same family of membrane proteins (GPCRs) as gut hormone receptors. Namely, beta-blockers’ on-targets are beta-adrenergic receptors from the class A GPCRs. What is more, both groups of receptors, beta-adrenergic receptors and incretin hormone receptors are expressed in the membrane of heart and vessels cells. Structure and sequence similarity together with similar cellular and tissue localization of those two groups of receptors are factors which make off-target interactions between beta-blockers and incretin hormone receptors likely. That is why we dedicated a separate study to those potential off-target interactions between beta-blockers and gut hormone receptors and described it in [25].

The main target (on-target) of another drug class—statins, which are well-known cholesterol-lowering agents, is HMG-CoA reductase which requires high glucose levels to be activated. HMG-CoA reductase is a transmembrane helical protein expressed in the membrane of endoplasmic reticulum with the statin binding site located in the cytosol C-terminal domain. It is known that statins can affect GPCR signaling, but rather through change in cholesterol levels and influence on the prenylation of the Rho family proteins [89] than a direct interaction with GPCRs. The mechanism of statin-induced diabetes [90, 91] is most probably linked to impaired insulin secretion and diminished insulin sensitivity [92, 93]. To our knowledge, no off-target interactions between statins and GPCRs have been confirmed experimentally. Yet, it was confirmed that simvastatin may block L-type Ca2+ channels, located in the cellular membrane like GPCRs, which regulate insulin secretion [8]. Moreover, a recent study [94] emphasized that statins can exhibit various pleiotropic effects in a cell, useful for drug repositioning [95, 96], especially atorvastatin [97] which was also among top VS hits discovered in our study (see the subsection: **New incretin therapies proposed by the in silico drug repositioning**). Therefore, we believe that the connection between statins and gut hormone GPCRs demonstrated by off-target interactions should not be excluded but certainly it needs the experimental confirmation.

As for the relative differences in binding affinities for three different incretin hormone receptors (GCGR vs. GIPR and GLP1R), they were rather insignificant. There is, however, an exception in the case of neurosteroids which were bound to GIPR stronger than to GCGR and GLP1R. Yet, neurosteroids were in general weakly bound to those receptors so that observation could be neglected. However, it is important to mention that GIPR models used in this study were based on GCGR receptor crystal structures (see Table 1). Yet, due to sequence similarity the best template for GIPR would be GLP1R. However, GLP1R crystal structures were not available at the time this study had been started. To test indisputably, whether the structural similarity of our GIPR models to GCGR structures influenced VS results, we computed Pearson correlation coefficients between XP-GScores obtained in VS with GIPR models and with GCGR and GLP1R models. On average, correlation coefficients between GIPR and GCGR were only slightly higher than between GIPR and GLP1R models. In the case of the orthosteric binding site, correlation coefficients were in the range of 0.49–0.81 (GCGR) and 0.48–0.80 (GLP1R) and in the case of the allosteric site in the range of 0.18–0.66 (GCGR) and 0.12–0.67 (GLP1R). Therefore, we believe that the bias introduced by using GCGR templates instead of GLP1R, although existing, could be ignored in this study.

### Diabetogenic and potential incretin effects demonstrated by drugs from SIDER

**Fig 3:**
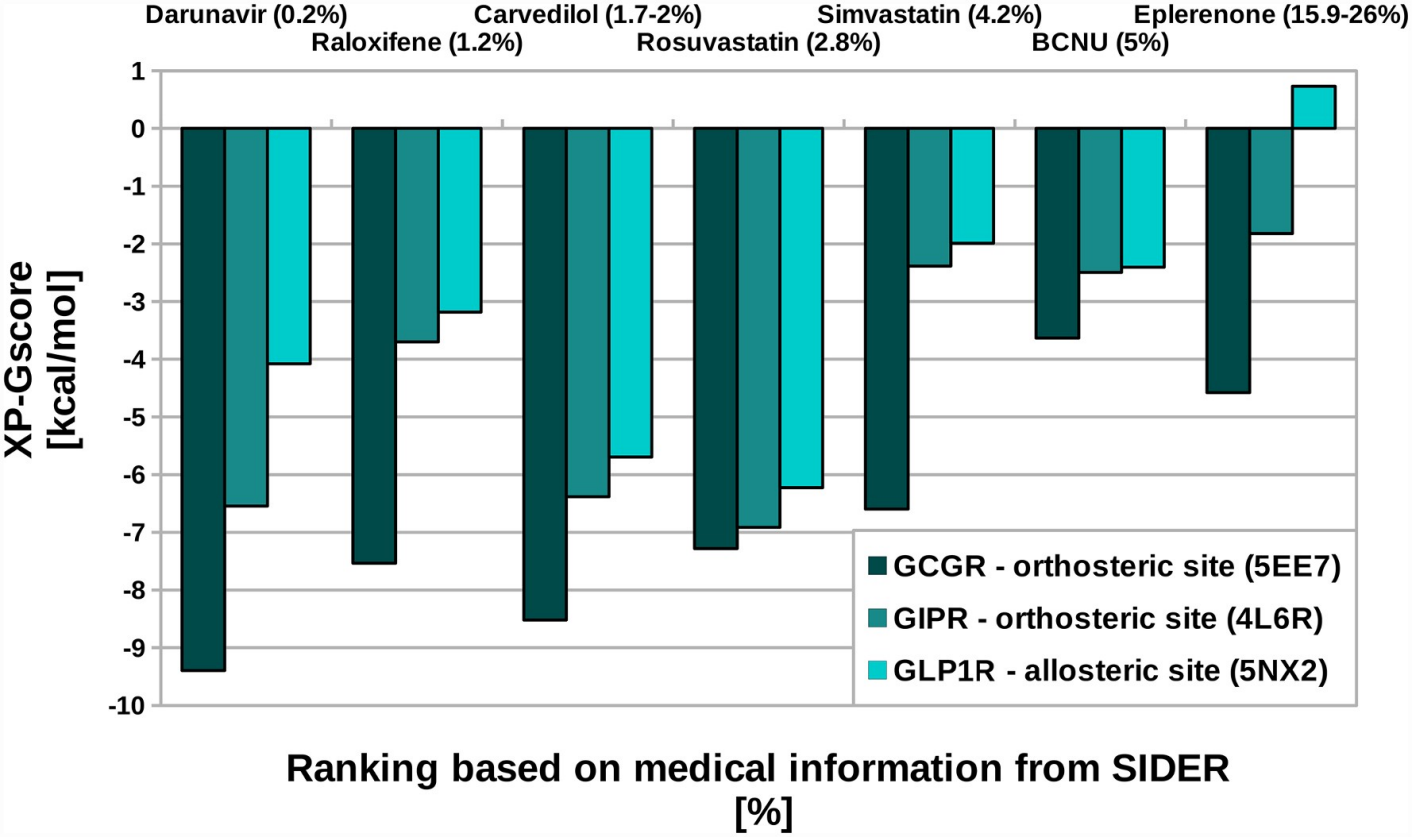
VS results for drugs selected from SIDER. Here, we presented values of XP-GScores for drugs selected from the SIDER database. Drugs were ranked with respect to the percentage of DM cases reported during the treatment. VS results for each gut hormone receptor were presented separately. For the sake of clarity, only one binding site for each receptor was presented here. We selected the binding site for which the Pearson correlation coefficient between the drug ranking based on XP-GScores and the drug ranking based on SIDER was the highest.

Although there is little data in the literature comparing drugs from different classes in the aspect of drug-induced T2DM, we managed to find such information, though highly limited, in SIDER [24]. SIDER (Side Effect Resource) is a database of side effects of drugs derived from clinical trials and medical literature. From SIDER we extracted (see Methods) data for several well-known drugs (see Table A in S1 File) which could be described as ‘small molecules’: darunavir, raloxifene, rosuvastatin, simvastatin, BCNU and eplerenone. Darunavir is an antiviral drug to treat AIDS, carvedilol belongs to beta-blockers, while rosuvastatin with simavastatin are statins. Raloxifene is used for treatment of osteoporosis. Eplerenone is a potassium-sparing diuretic which exhibits the antimineralocorticoid activity while BCNU (carmustine) is a chemotherapeutic. The medical information on the influence of those drugs on the glucose homeostasis was deposited in SIDER, i.e., in the form of the percentage of diabetes cases occurred during the pharmacotherapy. We converted that data into the ranking of those drugs (see Table A in S1 File). The best rank (1) was assigned to the drug which induced the lowest percentage of diabetes cases among all reported in SIDER drugs (darunavir). Again, as above (see Fig 2), we compared that ranking with results from the VS study using incretin hormone receptors structures (see Fig 3). And again, statins and beta-blockers together with darunavir exhibited the strongest binding affinity for gut hormone receptors and indeed were reported in SIDER as the least harmful among all reported glucose homeostasis disruptors. Based on that, we suggested that those strongly binding drugs among SIDER-derived drugs could owe their least T2DM-inducing ability to the compensating incretin effect which can regulate the glucose serum levels. Based on the current knowledge, such a beneficial side effect of those drugs cannot be discarded, yet should be certainly confirmed experimentally. In contrast, we observed also drugs (eplerenone and BCNU) that exhibited rather weak binding affinity for incretin hormone receptors (high values of XP-GScores). Most probably those drugs do not bind to incretin hormone receptors and consequently do not enhance the positive incretin effect which could compensate for their negative influence on the glucose serum level. Eplerenone and BCNU were both reported to jeopardize diabetic patients according to SIDER (see Table A in S1 File).

In addition to the above results, we computed Pearson correlation coefficients between drug-induced T2DM ranking derived from SIDER and the ranking based on XP-GScores derived from the VS results. The highest Pearson correlation coefficient between the SIDER-based ranking and the VS-based ranking in the aspect of drug-induced T2DM for all tested template structures and receptors was equal to 0.860 (GCGR, the orthosteric site), 0.724 (GLP1R, the allosteric site) and 0.713 (GIPR, the orthosteric site). We believe, that those high correlation coefficients confirm our hypothesis on the potential incretin effect, enhanced by off-target interactions with gut hormone receptors, which could compensate for other, T2DM-related side effects. As it was mentioned above, to enhance the incretin effect, a drug should stimulate GLP1R and GIPR and, on the contrary, inhibit GCGR. Yet, a more detailed computational study, involving both active and inactive conformations for each incretin receptor is needed to assess whether the suspected off-target interactions for drugs derived from SIDER are associated with the receptor stimulation or inhibition.

### Diabetogenic and potential incretin effects demonstrated by statins

**Fig 4:**
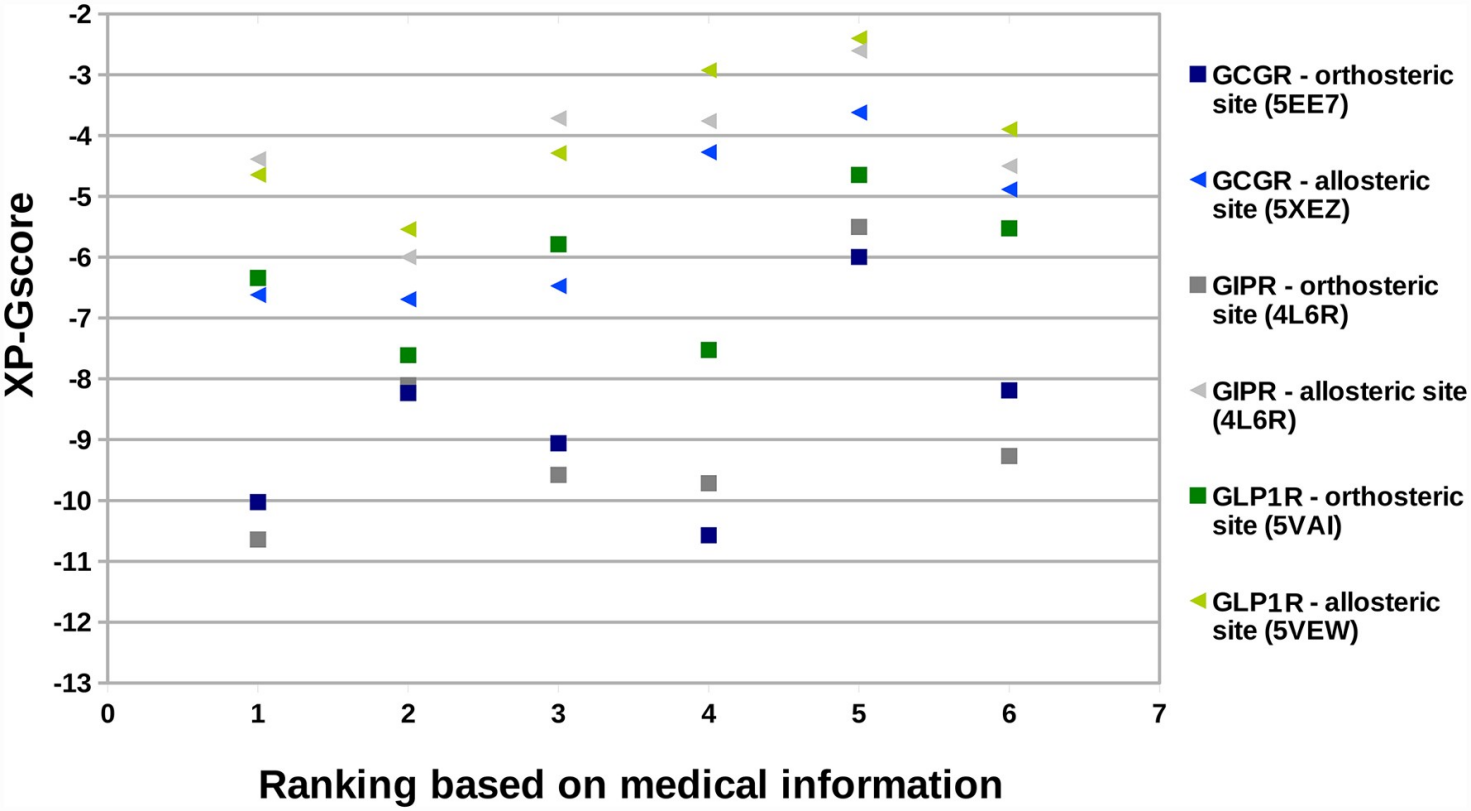
VS results for statins. Comparison of binding affinities (best XP-GScore) for the selected GPCRs with the drug ranking based on clinical trials involving diabetic patients. Assigned ranks according to [98]: 1 –pitavastatin, 2 –pravastatin, 3 –rosuvastatin, 4—atorvastatin, 5 –simvastatin, 6 –fluvastatin.

Only for statins and beta-blockers we managed to find in the literature the detailed, quantitative information on their relative diabetogenic effect. Based on that data, we prepared the T2DM-related ranking for all drugs from the statin drug class (see Table B in S1 File), as in the previous case of SIDER-derived drugs. We derived XP-GScores from VS using gut hormone receptors structures for all included statins and used it to prepare the ranking of their binding affinities for those selected GPCRs. Then, like in the case of data derived from SIDER, we computed Pearson correlation coefficients between the ranking of statins describing their T2DM-inducing risk and the ranking reflecting strength of their potential off-target interactions with gut hormone GPCRs. Comparing similar data for beta-blockers, described in our accompanying manuscript [25], the correlation was less significant, though still existing (see Fig 4). That may suggest that statins interact with incretin hormone receptors much weaker than beta-blockers or not all of them demonstrate such off-target interaction. Yet, still the relatively high Pearson correlation coefficient equal to 0.800 was observed, e.g., for the allosteric site of GCGR. In the case of GIPR and GLP1R the highest correlation coefficient was equal to 0.455 (allosteric) and 0.677 (allosteric), respectively. It is not to be missed, that the medical information on statin-induced diabetes is contradictory in some cases. For example, in [98] rosuvastatin was described as less harmful for the glucose homeostasis than simvastatin. The same observation was deposited in SIDER (see Table A in S1 File). However, in [99] it was stated that rosuvastatin had elevated glucose levels to the greater extent comparing to simvastatin. The above-mentioned clinical trials differ by the patient population included which could be the reason for such contradictory results. Interestingly, our approach successfully predicted that rosuvastatin can disturb the glucose serum levels to the smaller extent than simvastatin due to the potential enhancement of the compensating incretin effect. Namely, the binding affinity for both binding sites and to all gut hormone receptors of simvastatin was weaker comparing not only rosuvastatin but all included statins (see Fig 4). Interestingly, we observed the same result when using crystal structures of receptors for VS. In Figure A in S1 File we presented VS results for statins which were obtained using crystal structures of GCGR and GLP1R (PDB id: 5XEZ and 5VEW, respectively) and two MD-refined models (see Table E in S1 File) of those receptors instead of one best model like in Fig 4. Again, in all cases, rosuvastatin was bound to gut hormone receptors stronger than simvastatin regardless of slight differences between MD-refined and crystal receptor structures.

**Fig 5:**
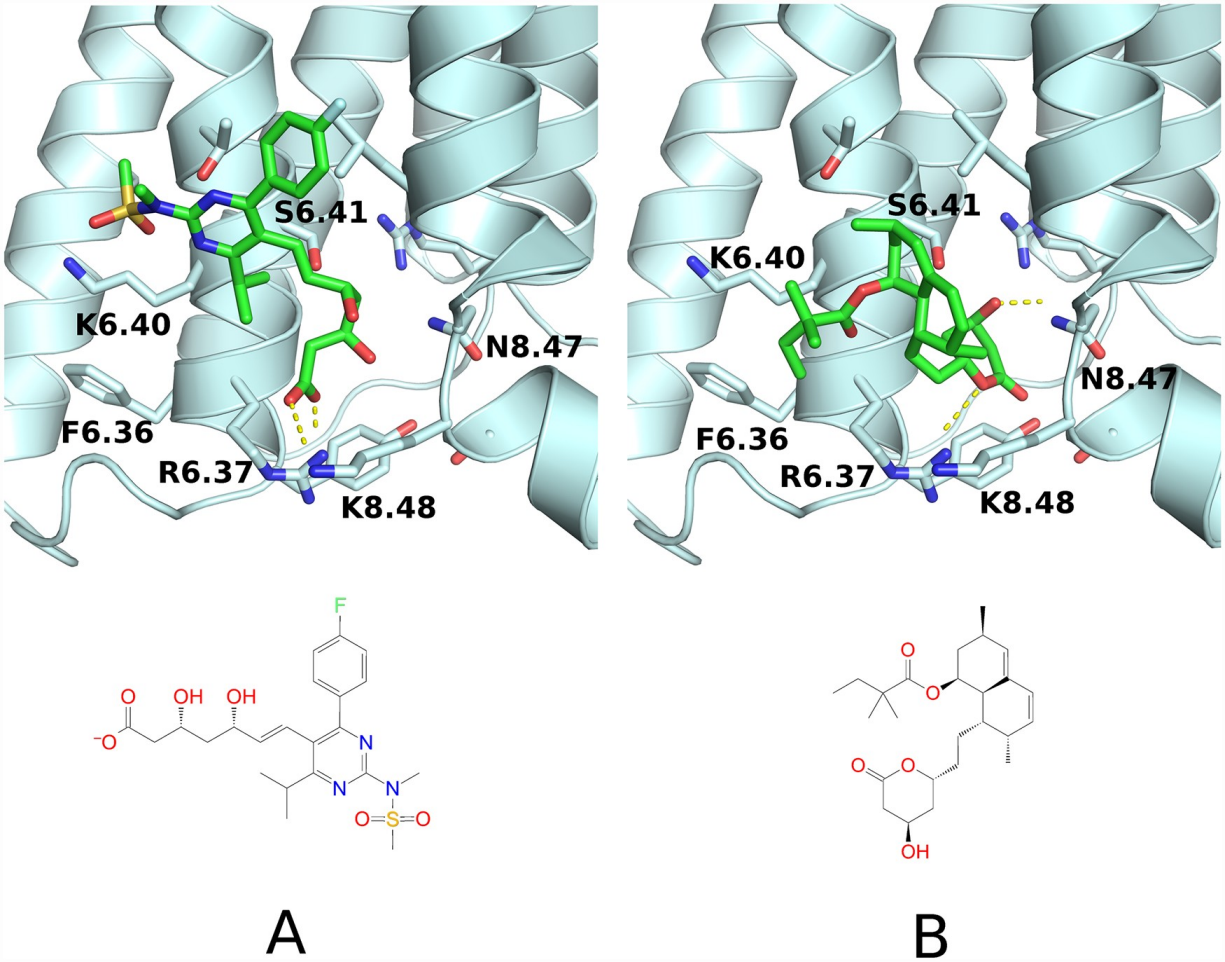
Rosuvastatin vs. simvastatin—The allosteric binding mode to GCGR predicted by Glide. (A) Binding mode of rosuvastatin—the allosteric site of GCGR. The salt bridge was formed between the carboxyl group of the statin and the amino group of the well-conserved R6.35 (B) Binding mode of simvastatin—the allosteric site of GCGR. Several polar contacts were formed with the receptor, e.g., with S6.41. Polar contacts were depicted with yellow dashed lines. The figure was prepared with Pymol [131].

Concerned with the above results, we examined binding modes of rosuvastatin and simvastatin in details. In Fig 5 we compared the top-ranked binding modes for two GCGR complexes with rosuvastatin (A) and simvastatin (B) inside the allosteric binding site. Both statins formed polar contacts with R6.35, but only in the case of rosuvastatin the salt bridge was formed due to its charged carboxyl group. We believe that this interaction could be crucial for differences in binding of those two statins. XP-GScores computed with Glide for the described binding modes were equal to -6.472 (rosuvastatin) and -3.620 (simvastatin).

**Fig 6:**
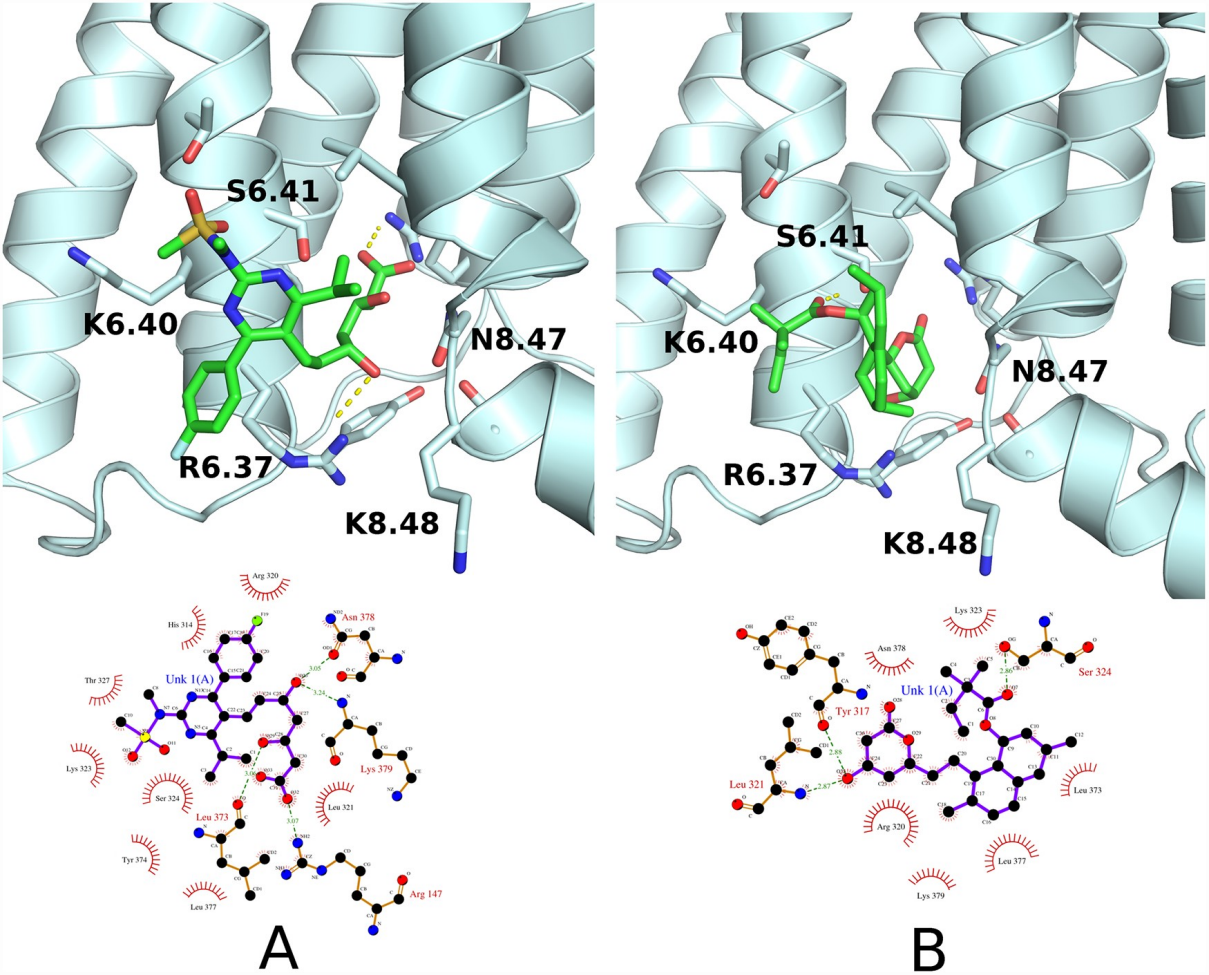
Rosuvastatin vs. simvastatin—The allosteric binding to GCGR predicted by Autodock VINA. Rosuvastatin (A) and simvastatin (B) binding modes were obtained with Autodock VINA implemented in GUT-DOCK. Here, we submitted to GUT-DOCK the mol2 files with ligands coordinates. Polar contacts with side chains were depicted with dashed yellow lines. Polar contacts with the main chain atoms were excluded for the sake of the figure clarity. Like presented in Fig 5, rosuvastatin formed polar contacts with R6.35, while simvastatin only with S6.41. The figure was prepared with Pymol [131] and Ligplot [132] implemented in GUT-DOCK.

We compared above results with the GUT-DOCK output (see Fig 6). In GUT-DOCK, ligands are flexibly docked to rigid receptors using Autodock VINA. Although for both statins most of their functional groups were located approximately in the same place as in Fig 5 some of them were rotated, e.g., a lactone ring of simvastatin was rotated by 180 degrees which resulted in the loss of a polar contact with Arg320 (R6.35). Autodock VINA provided the simvastatin binding mode with less polar contacts with GCGR than Glide, yet polar contacts with Ser324 (S6.41) were observed in both results. The rosuvastatin binding mode generated by Autodock VINA differed from the Glide-generated pose in the orientation of the fluorophenyl moiety. Autodock VINA (GUT-DOCK) located that functional group in the intracellular direction while Glide located it oppositely. Nevertheless, that phenyl ring was not involved in any interactions in both cases. On the contrary, the remaining N-methylmethanesulfonamid moiety of rosuvastatin was close to the Thr327 (T6.44) and Lys323 (K6.40) in both cases.

As for the theoretical binding energy, Autodock VINA docking scores for the presented ligand poses (see Fig 6) were surprisingly similar for rosuvastatin and simvastatin (-7.2 and -7.3, respectively) which did not reflect that rosuvastatin can bind gut hormone receptors stronger than simvastatin. We decided to rescore that binding modes with Glide to assess if the obtained binding energy is due to the binding modes their selves or to the force field. We used the ‘score in place’ option in Glide and similarly to Autodock VINA results we obtained: -1.739 (rosuvastatin) and -2.816 (simvastatin) which meant that both force fields provided the same result. We suspected that in this case, the heuristic algorithm of Autodock VINA for conformational search did not find the best-fitted ligand poses to the clinical data for both statins like Glide did. A comment is needed regarding the conformational search algorithm of Autodock VINA. Each step during the Broyden-Fletcher-Goldfarb-Shanno local optimization in Autodock VINA is accepted according to the Metropolis criterion. Consequently, each Autodock VINA simulation run starting with the system-generated random seed is slightly different. Yet, providing the sufficient number of simulation runs, the convergence of docking results should be finally reached [100]. Being aware of that, we examined the average score for all 10 poses generated with Autodock VINA instead of scores for only top-ranked poses, like described above. Indeed, rosuvastatin demonstrated better average binding energy than simvastatin (-6.99 comparing -6.46, respectively) in this case. What is more, rosuvastatin demonstrated more converged binding modes comparing simvastatin. Namely, the average RMSD of all 10 poses generated by Autodock VINA with respect to the top-ranked pose it was only 2.52 Å for rosuvastatin and 3.16 Å for simvastatin. As it was described in our previous study [101], not only the binding mode itself should be examined during the ligand docking but also the docking run convergence. In other words, high affinity ligands should not only be docked to a receptor with high scores (low binding energy values) but also the docking results should be converged which means that the standard deviations of RMSD values with respect to, e.g., the top-ranked pose should be rather small.

Pitavastatin, ranked as first and pravastatin ranked as second in both mentioned above clinical trials, are expected to be the least harmful for the glucose homeostasis among all statins (see Table B in S1 File). In our VS results both of those pharmaceuticals were of the strongest binding affinity for all incretin hormone receptors (the lowest values of XP-GScore). We believe our results on the statins drug class could suggest that at least in the case of statins of the strongest theoretical binding affinity for gut hormone receptors, pitavastatin, pravastatin and rosuvastatin, the compensating incretin effect on the glucose metabolism could indeed be demonstrated which could lead to the clinically observed decreased risk of T2DM. Certainly, our VS results should be confirmed with experimental studies. Yet, we believe that the current study is the first step in research on that topic and is sufficient to start such experimental studies.

### New incretin therapies proposed by the in silico drug repositioning

The last part of the current study was focused on a different aspect of gut hormone receptors. Namely, it was dedicated to *in silico* drug discovery targeting that distinct group of the class B GPCRs. Based on the concept of the drug repositioning we limited our investigations to pharmaceuticals which have been already registered in US (FDA-approved). We examined top-ranked in VS, compounds which could be used to improve the glucose metabolism by enhancing the incretin effect. VS top hits which were provided in Table 2 were not tested experimentally but were selected solely on the XP-GScore values. Thus, Table 2 may potentially include false-positive hits for the gut hormone receptors (e.g., contrast media) and should be examined with caution.

**Table 2:** Top hits from virtual screening for GCGR, GLP1R and GIPR receptors.

Name	Drug class / Target	The best XP-GScore [kcal/mol]
Valrubicin	Antibiotic	-12.758
Acarbose	Glycemic control / inhibitor of alpha glucosidase	-12.649
Paromomycin	Antibiotic	-12.050
Atorvastatin	Statin	-11.993
Iopromide	Contrast medium	-11.834
Pradaxa	Thrombin inhibitor	-10.975
Salmeterol	Beta-2 adrenergic receptor agonist	-10.673
Iohexol	Contrast medium	-10.664
Atazanavir	Antiretroviral drug	-10.581
Cangrelol	Antiplatelet drug	-10.418
Loversol	Contrast medium	-10.406
Ticagrelol	Platelet aggregation inhibitor	-10.241
Cobicistat	Antiretroviral drug	-10.193
Brexpiprazole	Dopamine D2 receptor partial agonist	-10.181
Ioxilan	Contrast medium	-10.010
Isavuconazonium	Antifungal drug	-9.975
Bimatoprost	Prostaglandin analog (ocular hypertension)	-9.936
Nebivolol	Beta-1 receptor blocker	-9.795
Ravicti	Urinary tract	-9.778
Aliskiren	Renin inhibitor	-9.719
Vilanterol	Beta-2 adrenergic receptor agonist	-9.595
Montelukast	Anti-asthma drug	-9.593
Cleocin	Antibiotic	-9.588
Lincomycin	Antibiotic	-9.574
Empagliflozin	Anti-diabetes drug	-9.564
Tirofiban	Antiplatelet drug	-9.494
Centany	Antibiotic	-9.473
Ospemifene	Selective estrogen receptor modulator	-9.421

As it was described above (see Fig 4), ligand poses obtained from VS using cavity-like orthosteric binding sites were better scored by XP-GScore than those targeting surface-like allosteric binding sites. Nevertheless, most of hit compounds (e. g., a small molecule ligand acarbose) were ranked high in both screenings—targeting orthosteric and allosteric sites, respectively. Acarbose is the FDA-approved drug used to treat diabetes type 2 [102]. Acarbose inhibits alpha glucosidase and it acts as a starch blocker delaying absorption of carbohydrates. Suggestion that gut hormone receptors could be off-targets for acarbose is plausible and highly beneficial. That would mean that the therapeutic action of acarbose on T2DM may be twofold: delaying absorption of carbohydrates and launching the incretin effect. Among known drug classes which are associated with drug-induced diabetes type 2 the highest rank was assigned to statins and beta-blockers: atorvastatin [103] and nebivolol (and salmeterol) (see Table 1). Other top hit compounds were antibiotics and antiviral drugs and also antiplatelet and anticoagulant drugs. Top hits discovered in this *in silico* study should be tested in vitro and in vivo to exclude false-positives.

## Conclusions

One of the directions of modern drug discovery is toxicology and side effects predictions. The current study and the accompanying study on beta-blockers [25], which was expanded by development of the web-service for small-molecule docking to the class B GPCRs (GUT-DOCK: http://gut-dock.miningmembrane.com), are guided towards that direction. Although more detailed, experimental studies, followed by years of thorough clinical observations of patients, are certainly needed to confirm our findings it is plausible that gut hormone receptors are off-target of many currently available pharmaceuticals as we showed in this study. Such off-target interactions of drugs enhancing the positive incretin effect could compensate for the negative influence of those drugs on the glucose serum level frequently leading to drug-induced T2DM. We believe that hypothetical compensation effect (stimulation of incretin GPCRs) could be used, e.g., in lead optimization to minimize T2DM-related side effects. To date, other ways of avoiding drug-induced T2DM are very few due to the fact that the exact molecular mechanism of such adverse reaction still remains too complex or unknown. Also, information derived from clinical trials is often contradictory, like shown in the case of statins. The major drawback of our study is a lack of experiments which could confirm our findings. Yet, such experiments, targeting gut hormone receptors are still of high cost and effort like in the case of any receptor belonging to the GPCR family. Only recently, gut hormone receptors have been characterized structurally by crystallography after long and tedious experimental studies. Although there are some experimental (and computational, e.g., ADME and ADME-TOX implemented in Schrodinger) methods for testing of drug bioavailability (its intestinal absorption, blood-brain permeability, unbound concentrations in serum and skin permeability) and pharmacodynamics they are not fitted explicitly to detect the off-target interactions. Interestingly, in the Schrodinger package, the concentration of unbound drug is predicted by computing its binding affinity for human serum albumin. Similarly, in our study, we tried to predict the drug effect on the glucose homeostasis by computing its binding affinity for GCGR, GIPR and GLP1R of GPCR family. Such off-target interactions can be valuable, e.g., when the drug acts as either a positive allosteric modulator or an agonist of GIPR and GLP1R, enhancing the incretin effect and improving the insulin secretion, or as a negative allosteric modulator or an antagonist of GCGR decreasing the glucose serum levels. Although CNS response to nutrients has been studied for some time [104], still little is known about gastrointestinal tract response to various pharmacotherapies. For that reason, we believe that our study brings valuable observations in the field of potential off-target interactions targeting gut hormone GPCRs which could help to improve the risk-benefit balance between the treatment effectiveness and developing new-onset diseases induced by pharmacotherapy.

## Methods

### The GPCR model building procedure

The current study had been started before releasing recent GLP1R crystal structures (see Table 1). For that reason, we included in the study also homology models of GLP1R and GIPR which were built using the glucagon receptor crystal structures (PDB id: 4L6R, 5EE7). There are only two templates representing an active conformation of GLP1R: 5NX2 and 5VAI and there are a few structures of an extracellular domain with a peptide ligand inside the orthosteric binding site (5NX2, 3IOL, 4ERS, 2QKH). In case of glucagon receptor only an inactive conformation was determined with X-ray. However, a recent molecular dynamics (MD) study [105] provided insights also into the active conformational state of GCGR and orientation and localization of its ECD domain together with a peptide agonist. That active GCGR model was obtained from the 2 microsec MD simulation starting from the homology model based on 4L6R (TMD) and 4ERS (ECD) PDB entries. Here, we used that GCGR model as a template to locate GIP and GLP peptides in the orthosteric binding sites of respective receptors homology models and to assess them with our program, described previously [25], which provided steric hindrance scores for every model.

Homology models of GIPR and GLP1R receptors were prepared with GPCRM [106] (see Fig 6) and our previously developed methodology for membrane proteins [101, 107–110]. A total number of 3000 models per each template/receptor pair were generated. 1500 models were discarded based on the steric hindrances scores to prevent congestion of the orthosteric binding site by extracellular loops. The remaining 1500 models were clustered using the Rosetta3.5 cluster application and evaluated with BCL::Score using knowledge-based potentials derived specifically for membrane proteins [111]. Five lowest energy models from five largest clusters were selected for the virtual screening study (VS).

GCGR and GLP1R crystal structures were used in the 20 ns MD simulation to generate an ensemble of receptor conformations. The structures of 4LR6, 5EE7, 5XEZ, 5VEW, and 5VEX contained only the transmembrane domain, the other structures contained additionally fragments of the extracellular domain. Each system, containing the protein embedded within the membrane and solvated, was prepared using the CHARMM-GUI Membrane Builder (http://www.charmm-gui.org) [112–115]. The membrane was formed of POPC and cholesterol molecules with proportion of 5:1. Each system was neutralized by addition of Na+ and Cl- ions, with ionic concentration of 0,15 M. Boundaries of the simulation box were set at least 15 Å away from protein atoms. The number of POPC and cholesterol molecules was equal to 100 and 20, respectively. The Charmm36 force field was used in each simulation [116]. The minimization stage included 2500 steps of steepest descent minimization followed by 2500 steps of conjugate gradient minimization. The force constant of 10.0 kcal mol^-1^ Å^-2^ was applied to all atoms of the protein (except P atoms of POPC and O3 atoms of cholesterol—with the force constant of 2.5 kcal mol^-1^ Å^-2^) to restrain their position during minimization. During the following six short runs of the equilibration process (first two runs– 100 ps in NVT, the next four runs of 100 ps in NPT), position restraints were gradually released. The Langevin thermostat was used to control temperature (310 K, the friction coefficient of 1.0 ps-1). The Berendsen barostat (1 bar) was used to control the external pressure. The time integration step was equal to 0.001 ps (first three runs), and then it was set to 0.002 ps. Hydrogen bonds were constrained using the SHAKE algorithm. The production run for each system lasted 20 ns. The GPU version of the pmemd module of AMBER14 package was used for all MD simulations [117]. Conformational fluctuations stabilized after about 10 ns of production runs and RMSD for the transmembrane helices region was equal to about 2.0 Å (see Figure A in S1 File). RMSD of the whole TMD was higher—about 2.2–3.3 Å because of the loops movement. We believe that observed slight structural changes were due to the fact that we removed ligands from the crystal structures which were used to build simulation systems. However, in our opinion, all MD simulations performed during this study were too short to observe significant structural changes leading to, e.g., unbound-ligand inactive conformations of receptors. By means of MD refinement simulations we only obtained a dynamic picture of each receptor structure yet still close to the crystal structure. RMSD observed for MD refinement simulations starting from the crystal structure which originally contained a ligand (PDB id: 5EE7, 5XEZ, 5VEW, 5VEX) did not differ substantially from RMSD observed for simulations starting from crystal structures without ligands (PDB id: 4L6R, 5NX2, 5VAI). For example, the highest RMSD fluctuations comparing the starting crystal structure was observed for 5VAI which did not contain any ligand but included a highly deformed by the receptor activation helix VI. Noteworthy, extending MD simulations could indeed change significantly binding sites resulting in the loss of characteristic structural features of studied receptors and worst enrichment factors (see **Filtering of GPCR models based on enrichment studies**). On the other hand, MD refinement simulations including ligands could result in obtaining very similar receptor conformations to their starting crystal structures. Such similar conformations would most probably provide in VS only such ligands which are similar to already known actives. 2000 snapshots per each structure were recorded (see Figures A and B in S1 File). 1000 structures were discarded based on the steric hindrances scores computed for the orthosteric binding site and the respective endogenous ligand (peptide). The procedure of the models filtering based on steric hindrances scores was described in our recent work [25]. The remaining 1000 structures were clustered and evaluated with BCL::Score. Five lowest energy structures from five largest clusters were selected for VS studies.

### Filtering of GPCR models based on enrichment studies

In the next step of our modeling pipeline GPCR models were evaluated in the first stage VS procedure (enrichment studies) to retrieve the best-performing in VS receptors structures, following a well-established approach described, e.g., in [118]. For that first stage VS we used a set of 10 known active ligands retrieved from BindingDB [119] and Pubchem databases [120] for each receptor (see [25]) and 500 decoys (50 for each active ligand) generated via the DUD-E website (http://dude.docking.org/) [121]. Each enrichment study was carried out with SP-Glide (Schrodinger) [122]. Two binding sites, orthosteric and allosteric (see Fig 1), were treated here separately.

Typical metrics were used for evaluation of VS results: EF1%, EF5%, EF10%, ROC, AUC and BEDROC(alpha = 20) [123]. Our results were comparable to results of the reference study describing the usage of the Schrodinger package in virtual screening [124]. Based on the enrichment factors, the best model was selected for each receptor and each binding site (see Table F in S1 File and results presented in our recent study [25]). In case of GLP1R active and inactive conformations were treated here separately. For each template/structure/active site two best-performing models (see Table F in S1 File and [25]) were selected for the final virtual screening on the ZINC15 FDA-approved database of compounds. There were two cases for GLP1R based on 5NX2 –with and without ECD domain. Structures without ECD domain did not produce satisfying EF values (data not shown) so we decided to use for VS the GLP1R model based on 5NX2 with ECD included. In our second manuscript [25], we compared the above results of EF study for MD-refined models with the EF study for crystal structures of GCGR and GLP1R receptors. We observed that with the currently available set of active ligands, crystal structures of both receptors were not superior to MD-refined models in retrieving actives from non-binding decoys.

### Virtual screening against the ZINC15 FDA-approved drug library

GPCR models selected with the above VS procedure were used in the second stage VS of the ZINC15 database of ready-to-dock compounds [125]. We used SDF files belonging to a subset of ZINC15 which included coordinates of drug compounds approved by Food and Drug Administration (FDA) in USA. Additionally, we included several drugs which were not in the FDA-approved subset but were important for the current study because they belonged to the statin, diuretics or steroid drug class approved in Europe. Those additional ligands were downloaded in the SDF format from other subsets of ZINC15 (http://zinc15.docking.org/) or from the PubChem database (https://pubchem.ncbi.nlm.nih.gov/). All SDF files with ligands coordinates were prepared with LigPrep [126]. Multiple conformations per each ligand were kept providing finally a total number of 2240 ligand conformations for VS. XP-GScore was used to rank docked compounds [127]. We kept multiple ZINC15 entries for one active compound, if existing, e.g., ZINC000001530639 and ZINC000001886617 for fluvastatin (see Figure A in S1 File). As we observed in that example, such multiple ZINC15 entries included different conformational states of an active compound and thus docking results could be different. In Figure A in S1 File we presented VS results using crystal structures and MD-refined models of GCGR and GLP1R. In the case of crystal structures we presented results for ZINC000001886617 (a trade name Lescol) and ZINC000001530639 separately. In most cases, for the GLP1R crystal structure and for MD-refined models of GCGR and GLP1R (data not shown), we observed slight differences (less than 0.5) in values of XP-GScore with Lescol poses always ranked worse. Yet, in one case—VS using a crystal structure of GCGR (see Figure A in S1 File), we observed a significant difference (more than 3) in values of XP-GScore with again the Lescol pose ranked worse than the ZINC000001530639 pose.

Receptors grids were centered on the terminal peptide residue in the case of the orthosteric binding sites or on the NAM position derived from the GCGR crystal structure (5XEZ). Crystal structures of GLP1R 5NX2 and 5VAI did not include allosteric modulators, so the receptor model was aligned to 5XEZ prior to the grid generation. However, such definition of the receptor grid box center provided (most probably) some failed results in virtual screening. Namely, resulting ligands poses were docked inside TMD close to the intracellular part of the receptor instead of being docked to the allosteric binding site defined in crystallographic studies of GCGR. Therefore, we manually moved the receptor grid to face it more to the membrane rather than inside of TMD. Such settings provided VS results with less satisfying XP-GScores (the best ligand pose with XP-GScore equal to -8.866 instead of -13.957) but all ligands were properly docked to the allosteric binding site facing the membrane. We believe that the above difference in docking scores is due to limitations of Glide which do not include the membrane-specific energy terms during the docking. To analyze VS results we used the ‘Select top poses’ functionality in Schrodinger with maximum 50 poses as an output and all poses generated in this study for GCGR, GIPR and GLP1R (both, the orthosteric and allosteric binding sites together).

### Extracting medical information on diabetes mellitus from SIDER

To extract information of the relative diabetogenic effect of various drugs from SIDER we searched that database with the MeDRA preferred term: ‘diabetes mellitus’ (DM). That produced 176 drug entries, out of which only 16 had quantity (percentage of cases) rather than quality (rare, uncommon, postmarketing, infrequent, etc.) description of occurrence of that side effect among patients. Out of those 16 drugs we selected 7 which were of low molecular weight, acceptable for the small-molecule docking procedure. Thus, we excluded peptide analogs such as goserelin and Signifor or a cyclic everolimus and tacrolimus. We also excluded Methotrexate but for other reason. Namely, the percentage range associated with the number of DM cases among patients was equal to: 1–10%. Such broad, imprecise range was difficult to be interpreted into a relative rank with respect to the rest of drugs selected from SIDER. Although T2DM-oriented clinical trials are still being carried out providing new medical data, SIDER is also constantly updated (see corresponding manuscripts published in 2008 [128], 2010 [129] and 2016 [24]) and linked with another database of chemical-protein interaction network STITCH [130].

## Supporting information

S1 File Supplementary_material.pdf. Supplementary material. A file including: tables with referenced medical information on drug-induced diabetes, figures presenting the modeling procedure, MD refinement simulations of GCGR and GLP1R, the docking procedure in Glide.
(PDF)

S2 File Data_set_file.xlsx—A file including data generated during the current study. (XLSX)

## Abbreviations

ADME: absorption, distribution, metabolism, and excretion
ECD: extracellular domain
GCGR: glucagon receptor
GIPR: gastric inhibitory polypeptide receptor
GLP1R: glucagon-like peptide-1 receptor
GPCR: G protein-coupled receptor
MD: molecular dynamics
TMD: transmembrane domain
VS: virtual screening
