# Supplementary material for: Drug-induced diabetes type 2: In silico study involving class B GPCRs
Source: PLoS One. 2019 Jan 16;14(1):e0208892. doi: 10.1371/journal.pone.0208892 (PMC6334951; doi:10.1371/journal.pone.0208892)
Supplement: S1 File — Supplementary material. A file including: tables with referenced medical information on drug-induced diabetes, figures presenting the modeling procedure, MD refinement simulations of GCGR and GLP1R, the docking procedure in Glide. (PDF) [file pone.0208892.s001.pdf]

Drug-induced diabetes type 2: in silico study involving class B GPCRs.

Dorota Latek, Ewelina Rutkowska, Szymon Niewieczerzal, Judyta Cielecka-Piontek

Supplementary material

A file including: tables with referenced medical information on drug-induced diabetes, figures presenting the modeling procedure, MD refinement simulations of GCGR and GLP1R, the docking procedure in Glide.

Table A. Clinical data on drug-induced diabetes and glucose homeostasis disruption derived from SIDER.

Name	Rank based on SIDER [1]	Percentage range of T2DM-related side-effects occurrence based on SIDER
darunavir	1	0.2%
raloxifene	2	1.2%
carvedilol	3	1.7-2%
rosuvastatin	4	2.8%
simvastatin	5	4.2%
BCNU	6	5%
eplerenone	7	15.9-26%

Table B. Ranking of statins based on the medical information from two clinical trials on drug-induced diabetes.

Name	Rank based on clinical trial no 1 [2]	Rank based on clinical trial no 2 [3]
pitavastatin	1	No data

pravastatin	2	2
rosuvastatin	3	5
atorvastatin	4	4
simvastatin	5	3
fluvastatin	6	1

Table C. Ranking of diuretics based on the medical information from clinical trials on drug-induced diabetes.

Name	Diuretic class	Rank based on clinical trials [4-10]
methazolamide	Carbonic anhydrase inhibitor	1
acetazolamide	Carbonic anhydrase inhibitor	1
eplerenone	Potassium-sparing diuretic	2
triamterene	Potassium-sparing diuretic	2
spironolactone	Potassium-sparing diuretic	2
mefruside	Thiazide analog	3
indapamide	Thiazide analog	4
chlortalidone	Thiazide analog	5
clopamide	Thiazide analog	6
torasemide	Loop diuretic	7
furosemide	Loop diuretic	8
bumetanide	Loop diuretic	9
bendroflumethiazide	Thiazide	10
hydrochlorothiazide	Thiazide	11

Table D. Assignment of selected drugs to the steroid drug class.

Name	Steroid type
deflazacort	Glucocorticosteroid
prednisone	Glucocorticosteroid
betamethasone	Glucocorticosteroid
dexamethasone	Glucocorticosteroid
methylprednisolone	Glucocorticosteroid
fluorometholone	Glucocorticosteroid
hydrocortisone	Glucocorticosteroid
prednisolone	Glucocorticosteroid
triamcinolone	Glucocorticosteroid
cortisone	gluco/mineralocorticosteroid
aldocorten	Mineralocorticosteroid
fludrocortisone	Mineralocorticosteroid
progesteron	Neurosteroid
DHEA	Neurosteroid
ganaxolone	Neurosteroid
allopregnanolone	Neurosteroid
4-androstadienol	Neurosteroid

Table E. Results of enrichment studies.

Receptor	Template PDB id / binding site<br>Model number	BEDROC<br>(alpha=20)	ROC	AUC	EF1%	EF5%	EF10%
GLP1R	5ee72 - allosteric						
	Model 1	0.601	0.95	0.94	38	14	7
	Model 2	0.680	0.99	0.99	29	16	8
	5nx2 (ECD included) - orthosteric						

	Model 1	0.23	0.91	0.91	0	6.1	5
	Model 2	0.23	0.91	0.91	0	4	5
	5nx2 - allosteric						
	Model 1	0.556	0.98	0.98	19	12	8
	Model 2	0.554	0.94	0.93	38	12	6
	5vai - orthosteric						
	Model 1	0.478	0.95	0.94	38	8.1	6
	Model 2	0.585	0.93	0.93	38	12	7
	5vew - allosteric						
	Model 1	0.589	0.94	0.94	29	14	7
	Model 2	0.559	0.98	0.97	38	10	8
	5vew - orthosteric						
	Model 1	0.423	0.93	0.92	0	12	6
	Model 2	0.330	0.93	0.92	9.6	6.1	5
GIPR							
	4l6r - orthosteric						
	Model 1	0.773	0.94	0.93	50	18	8.9
	Model 2	0.668	0.95	0.95	40	14	7
	4l6r - allosteric						
	Model 1	0.453	0.94	0.94	10	12	8.9
	Model 2	0.518	0.92	0.91	30	10	7
GCGR	5xez - orthosteric						
	Model 1	0.741	0.91	0.91	43	16	8
	Model 2	0.440	0.89	0.89	11	10	6
	5xez - allosteric						
	Model 1	0.441	0.90	0.89	0	12	8
	Model 2	0.419	0.89	0.88	11	10	7

4l6r - orthosteric						
Model 1	0.496	0.89	0.88	32	10	6
Model 2	0.327	0.80	0.79	11	6.1	5
5ee7- orthosteric						
Model 1	0.633	0.90	0.89	43	12	7
Model 2	0.454	0.88	0.88	32	8.1	5
4l6r - allosteric						
Model 1	0.540	0.92	0.92	32	12	6
Model 2	0.520	0.89	0.88	32	10	6
5ee7 - allosteric						
Model 1	0.817	0.94	0.94	43	18	8
Model 2	0.668	0.93	0.92	21	14	9

Results provided in this table were partly adapted from [11]

Table F. Active compounds used for enrichment studies.

Receptor	CHEMBL ID	SMILES
GCGR	CHEMBL3673123	C1=CC=C(C(=C1)CNC2=CC=C(C=C2)C3=CC=C(C=C3)Cl)C4=CC=C(C=C4)C(=O)NCCC(=O)O
	CHEMBL1933354	CCCC(C1=CC=C(C=C1)C(=O)NCCC(=O)O)OC2=CN(N=C2)C3=CC=CC(=C3)CC
	CHEMBL1933348	CCCC1=C(C(=C(C=C1C(C)C)C(C)C)C(C)O)C2=CC=C(C=C2)F
	CHEMBL1933361	CCCC(C1=CC=C(C=C1)C(=O)NCCC(=O)O)OC2=CN(N=C2)C3=NC=C(C=C3)C(F)(F)F
	CHEMBL3799802	CC(C1=CC=C(C=C1)C(=O)NCCC(=O)O)N2C(=CC(=N2)C3=CC(=CC(=C3)Cl)Cl)C4=CC5=C(C=C4)C=C(C=C5)OC
	CHEMBL486634	C1CCC(=CC1)C2=CC=C(C=C2)N(CC3=CC=C(C=C3)C(=O)NCC(C(=O)O)O)C(=O)NC4=CC(=CC(=C4)Cl)Cl
	CHEMBL1644183	C1=CC(=CC=C1CN2C(=CC(=N2)C3=CC(=CC(=C3)Cl)Cl)C4=CC=C(C=C4)OC(F)(F)F)C(=O)NCCC(=O)O

	CHEMBL1644178	CC(C)(C)C1CCC(CC1)C2=CC(=NN2CC3=CC=C(C=C3)C(=O)NC4=NNN=N4)C5=CC=C(C=C5)OC(F)(F)F
	CHEMBL1644180	C1=CC(=CC=C1CN2C(=CC(=N2)C3=CC=C(C=C3)OC(F)(F)F)C4=CC=C(C=C4)OC(F)(F)F)C(=O)NC5=NNN=N5
	CHEMBL1933358	CCCC(C1=CC=C(C=C1)C(=O)NCCC(=O)O)OC2=CN(N=C2)C3=CC(=CC=C3)OC
GIPR	CHEMBL446821	C1CC2=C(C1)C=C(C=C2)N(CC3=CC=C(C=C3)C(=O)NCCC(=O)O)C4=NC(=CS4)C5=CC=C(C=C5)OC(F)(F)F
	CHEMBL501628	C1=CC(=CC=C1CN(C2=CC=C(C=C2)OC(F)(F)F)C3=NC(=CS3)C4=CC=C(C=C4)C(F)(F)F)C(=O)NCCC(=O)O
	CHEMBL62444	CC(C)(C)C1CCC(CC1)N(CC2=CC=C(C=C2)C(=O)NCCC(=O)O)C(=O)NC3=CC=C(C=C3)OC(F)(F)F
	CHEMBL232224	CC(C)(C)C1CCC(CC1)N(C2CCC3=C2C=CC(=C3)C(=O)NCCC(=O)O)C(=O)CC4=CC=C(C=C4)OC(F)(F)F
	CHEMBL411832	CC(C)(C)C1CCC(CC1)N(C2CCOC3=C2C=CC(=C3)C(=O)NC4=NNN=N4)C(=O)NC5=CC=C(C=C5)OC(F)(F)F
	CHEMBL452067	CC1=CC(=CC(=C1)NC(=O)N(CC2=CC=C(C=C2)C(=O)NCC(C(=O)O)O)C3CCC(CC3)C(C)(C)C)C
	CHEMBL452311	C1CCC(CC1)C2=CC=C(C=C2)N(CC3=CC=C(C=C3)C(=O)NCC(C(=O)O)O)C(=O)NC4=CC(=CC=C4)Br
	CHEMBL456738	COC1=CC(=CC(=C1)NC(=O)N(CC2=CC=C(C=C2)C(=O)NCC(C(=O)O)O)C3=CC=C(C=C3)C4CCCCC4)C(F)(F)F
	CHEMBL453457	C1CCC(CC1)C2=CC=C(C=C2)N(CC3=CC=C(C=C3)C(=O)NCC(C(=O)O)O)C(=O)NC4=CC=C(C=C4)SC(F)(F)F
	CHEMBL1922839	CCCOC1=CC(=CC2=C1N(C(=NC3=CC=C(C=C3)C(C)(C)C)N2CC4=CC=C(C=C4)C(=O)NC5=NNN=N5)C)C(F)(F)F
GLP1R	CHEMBL198736	CC(C)(C)C1CCC(CC1)N(CC2=CC=C(C=C2)C(=O)NCCC(=O)O)C(=O)NC3=CC=C(C=C3)OC(F)(F)F
	CHEMBL219384	CC(C)(C)C1=CC=C(C=C1)N(CC2=CC=C(C=C2)C(=O)NCCC(=O)O)C(=O)NC3=CC=C(C=C3)OC(F)(F)F
	CHEMBL386446	C1CCC(=CC1)C2=CC=C(C=C2)N(CC3=CC=C(C=C3)C(=O)NCCC(=O)O)C(=O)NC4=CC(=CC(=C4)Cl)Cl
	CHEMBL487476	C1CCC(=CC1)C2=CC=C(C=C2)N(CC3=CC=C(C=C3)C(=O)NCC(C(=O)O)O)C(=O)NC4=CC(=CC(=C4)Cl)Cl

CHEMBL519903	C1CCC(=CC1)C2=CC=C(C=C2)N(CC3=CC=C(C=C3)C(=O)NCC(C(=O)O)O)C(=O)NC4=CC(=CC(=C4)Cl)Cl
CHEMBL499160	CC(C)(C)C1CCC(CC1)N(CC2=CC=C(C=C2)C(=O)NCC(C(=O)O)O)C(=O)NC3=CC(=CC(=C3)C(F)(F)F)C(F)(F)F
CHEMBL452310	COC(CNC(=O)C1=CC=C(C=C1)CN(C2=CC=C(C=C2)C3=CCCCC3)C(=O)NC4=CC(=CC(=C4)Cl)Cl)C(=O)O
CHEMBL1933365	C1CCC(C1)C(C2=CC=C(C=C2)C(=O)NCCC(=O)O)NC3=CN(N=C3)C4=CC=C(C=C4)C(F)(F)F
CHEMBL1933360	CCCC(C1=CC=C(C=C1)C(=O)NCCC(=O)O)OC2=CN(N=C2)C3=CC=C(C=C3)C(F)(F)F
CHEMBL198736	CC(C)(C)C1CCC(CC1)N(CC2=CC=C(C=C2)C(=O)NCCC(=O)O)C(=O)NC3=CC=C(C=C3)OC(F)(F)F

Figure A. VS results for statins – impact of the receptor conformation. Here, we compared VS results obtained with two MD-refined models and one crystal structure for each receptor (GCGR (A) and GLP1R (B)). There were two entries for two different conformations of fluvastatin (ZINC000001530639 – fluvastatin (C) and ZINC000001886617 – Lescol (D)) in the FDA-approved subset of ZINC15. We included both of them in VS and presented results for each of the conformers individually (see (A) and (B)). In all cases, also in the case of MD-refined models (data not shown) Lescol was bound to gut hormone receptors weaker than fluvastatin. Images presented in (C) and (D) were extracted from ZINC15.

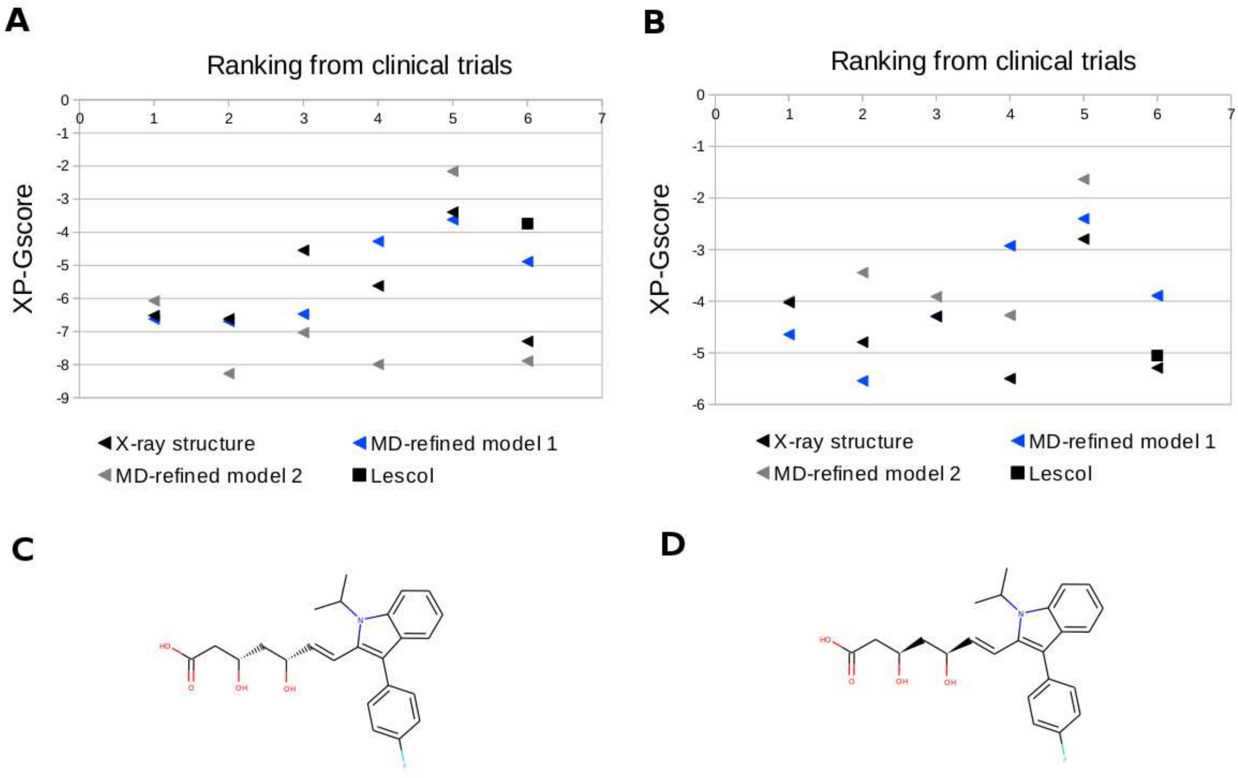

Figure B. The modeling procedure. Here, we showed the schematic pipeline of the model building and virtual screening procedures used in the current study.

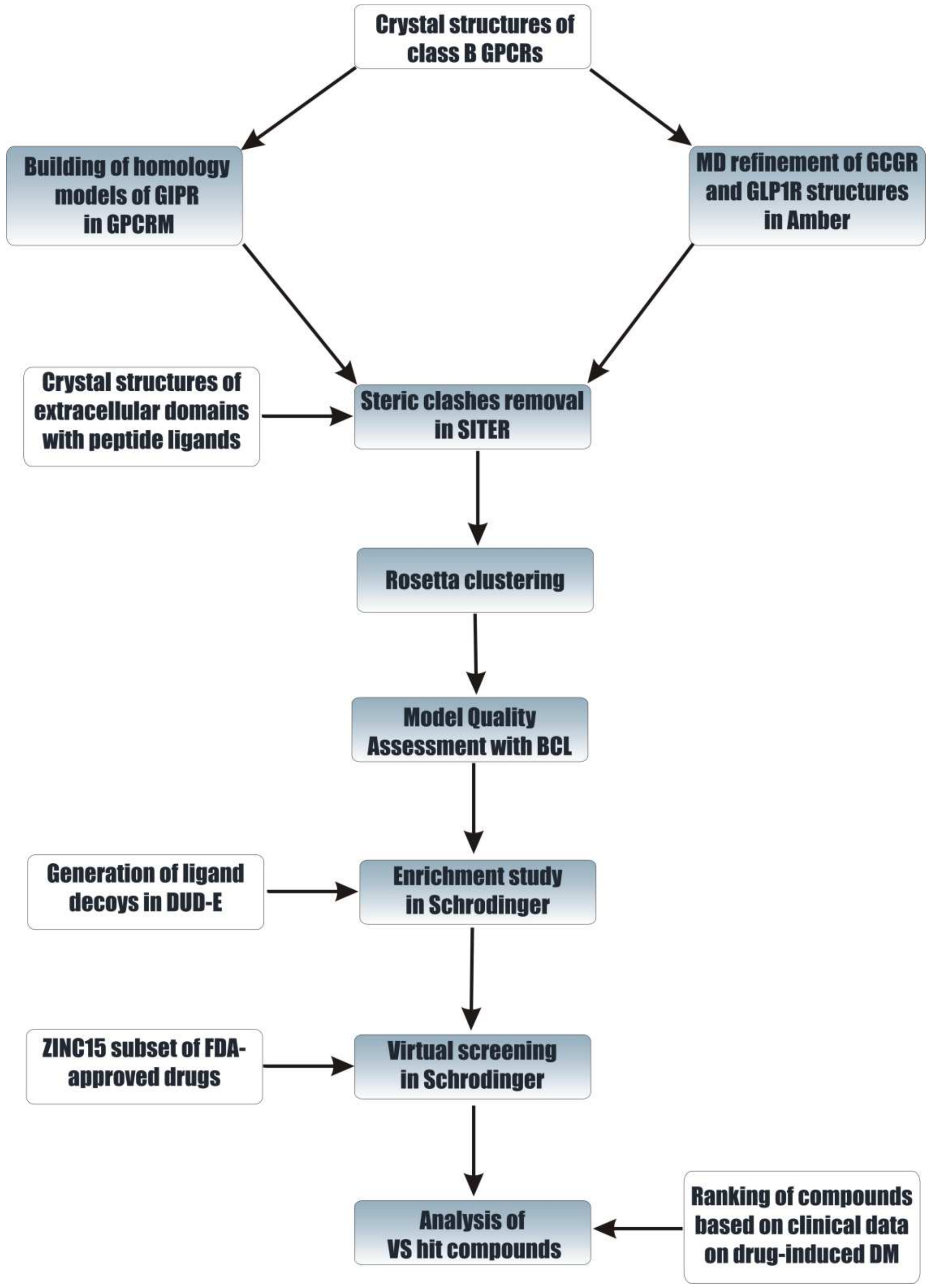

Figure C. Results of MD refinement simulations of GCGR. Root mean square deviation curves of the transmembrane helical core for GCGR homology models: (A) 4L6R, (B) 5EE7, and (C) 5XEZ, collected in equilibrium MD simulations, partly adapted from [11].

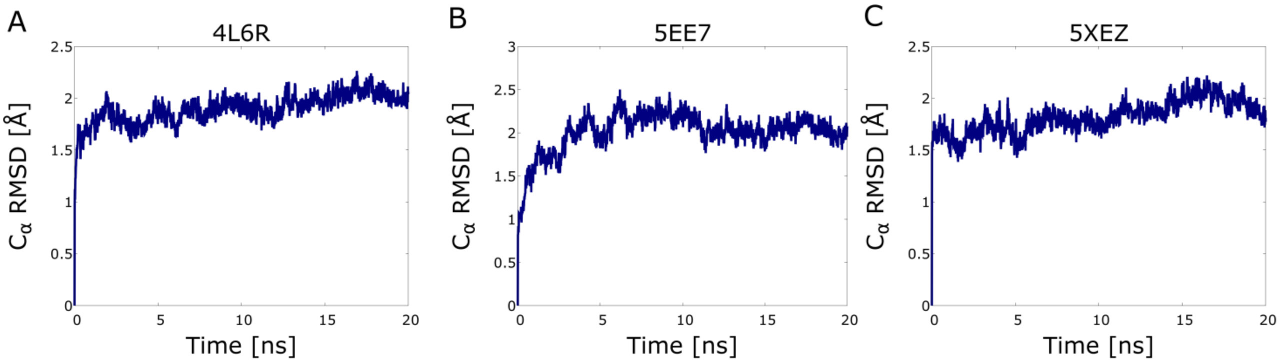

Figure D. Results of MD refinement simulations of GLP1R. Root mean square deviation curves of the transmembrane helical core for GLP1R homology models: (A) 5NX2, and (B) 5VAI, (C) 5VEW and (D) 5VEX, collected in equilibrium MD simulations. Starting structures in (A) and (B) represent the active form of the receptor, and inactive form in (C) and (D). Results for (C) were partly adapted from [11].

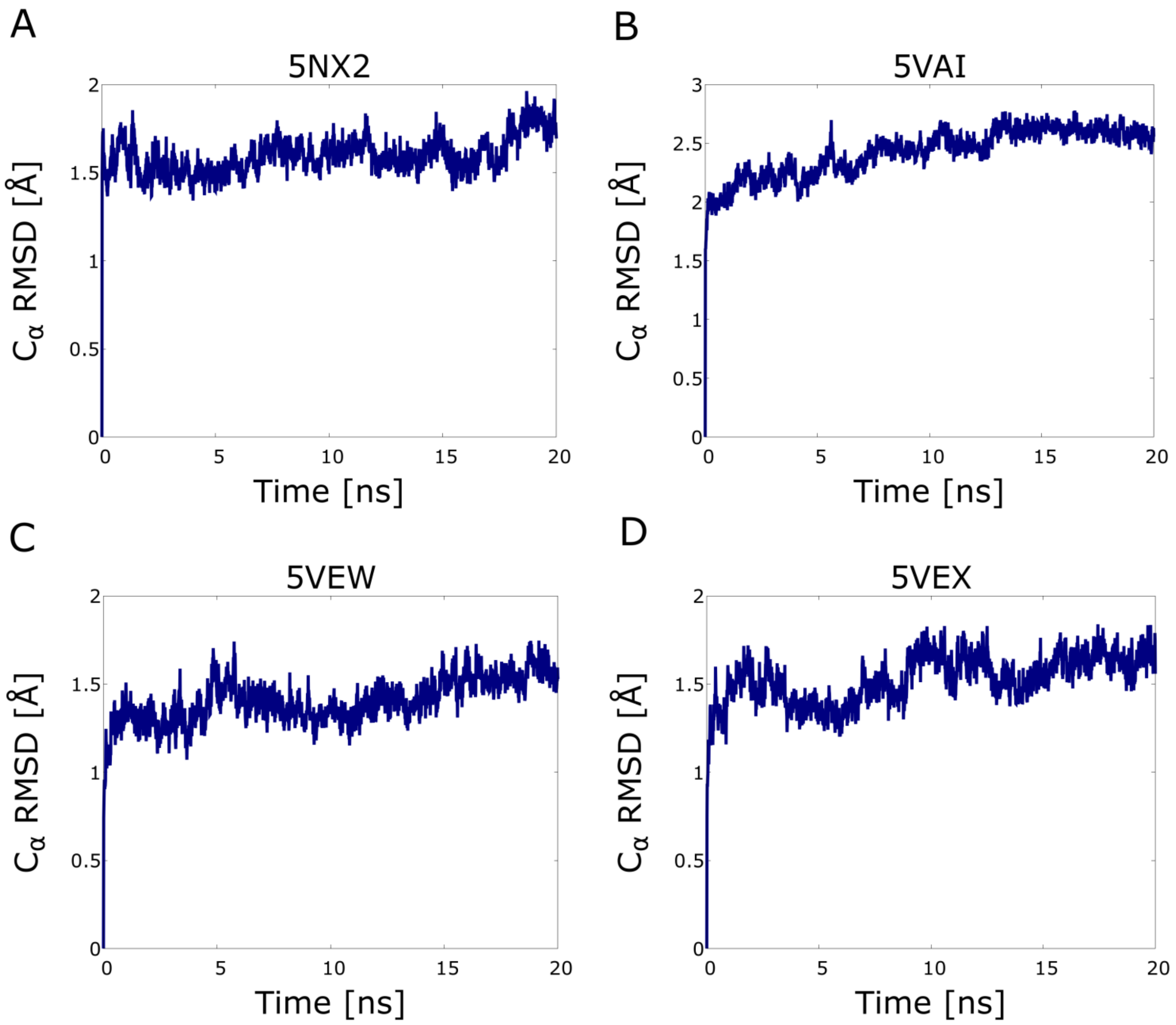

Figure E. Definition of the docking boxes in Glide. Here, outer diameter of both boxes located in the orthosteric (A) and allosteric (B) sites of GLP1R is equal to 30 Å. Both boxes cover nearly half of the receptor. To compare, diameter of PF-06372222 (antagonist molecule in a complex with GLP1R deposited in the 5VEW entry) is ca. 14 Å.

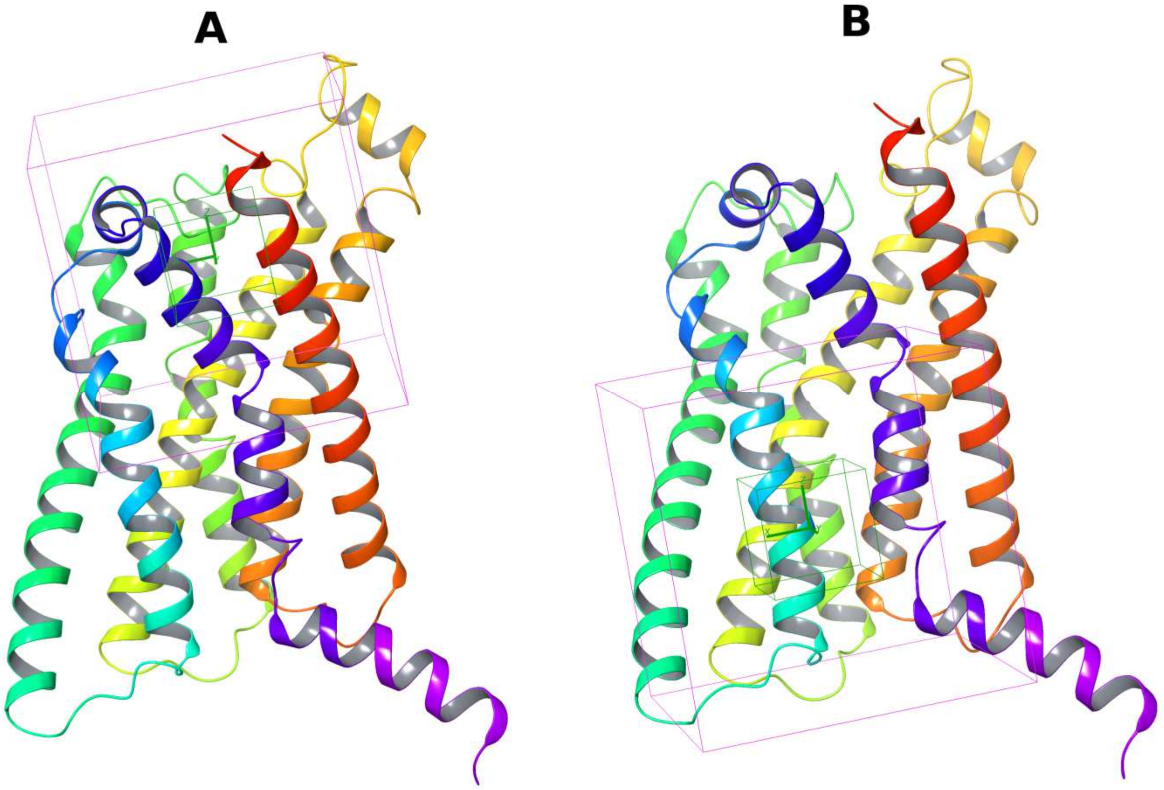

References

1. Kuhn M, Letunic I, Jensen LJ, Bork P. The SIDER database of drugs and side effects. Nucleic acids research. 2016;44(D1):D1075-9. doi: 10.1093/nar/gkv1075. PubMed PMID: 26481350; PubMed Central PMCID: PMC4702794.

2. Yoon D, Sheen SS, Lee S, Choi YJ, Park RW, Lim HS. Statins and risk for new-onset diabetes mellitus: A real-world cohort study using a clinical research database. Medicine.

2016;95(46):e5429. doi: 10.1097/MD.0000000000005429. PubMed PMID: 27861386; PubMed Central PMCID: PMC5120943.

3. Carter AA, Gomes T, Camacho X, Juurlink DN, Shah BR, Mamdani MM. Risk of incident diabetes among patients treated with statins: population based study. Bmj. 2013;346:f2610.

4. Hirst JA, Farmer AJ, Feakins BG, Aronson JK, Stevens RJ. Quantifying the effects of diuretics and β-adrenoceptor blockers on glycaemic control in diabetes mellitus–a systematic review and meta-analysis. British journal of clinical pharmacology. 2015;79(5):733-43.

5. Arumugam S, Sreedhar R, Miyashita S, Karuppagounder V, Thandavarayan RA, Giridharan VV, et al. Comparative evaluation of torasemide and furosemide on rats with streptozotocin-induced diabetic nephropathy. Experimental and molecular pathology. 2014;97(1):137-43.

6. Sica DA. Diuretic-Related Side Effects: Development and Treatment. The Journal of Clinical Hypertension. 2004;6(9):532-40.

7. Bozkurt Ö, De Boer A, Grobbee DE, De Leeuw PW, Kroon AA, Schiffers P, et al. Variation in renin–angiotensin system and salt-sensitivity genes and the risk of diabetes mellitus associated with the use of thiazide diuretics. American journal of hypertension. 2009;22(5):545-51.

8. Konstantopoulos N, Molero JC, McGee SL, Spolding B, Connor T, de Vries M, et al. Methazolamide is a new hepatic insulin sensitizer that lowers blood glucose in vivo. Diabetes. 2012;61(8):2146-54.

9. Dimitriadis G, Leighton B, Parry-Billings M, Tountas C, Raptis S, Newsholme EA. Furosemide decreases the sensitivity of glucose transport to insulin in skeletal muscle in vitro. European journal of endocrinology. 1998;139(1):118-22.

10. Preiss D, Veldhuisen DJ, Sattar N, Krum H, Swedberg K, Shi H, et al. Eplerenone and new-onset diabetes in patients with mild heart failure: results from the Eplerenone in Mild Patients

Hospitalization and Survival Study in Heart Failure (EMPHASIS-HF). European journal of heart failure. 2012;14(8):909-15.

11. Pasznik P, Rutkowska E, Niewieczerzal S, Cielecka-Piontek J, Latek D. Potential off-target effects of beta-blockers on gut hormone receptors: in silico study including GUT-DOCK - a web service for small-molecule docking. PloS one. 2019;Forthcoming.
